# Atomistic Insights into the Origin of High‐Performance Thermoelectric Response in Hybrid Perovskites

**DOI:** 10.1002/advs.202300666

**Published:** 2023-05-11

**Authors:** Wen Shi, Mingjia Yao, Xiaomei Wu, Tingxia Zhou, Xue Yong, Tianqi Deng, Huili Ma, Jinyang Xi

**Affiliations:** ^1^ School of Chemistry Sun Yat‐sen University Guangzhou 510006 China; ^2^ Materials Genome Institute Shanghai University Shanghai 200444 China; ^3^ Department of Chemistry The University of Sheffield Brook Hill Sheffield S3 7HF UK; ^4^ State Key Laboratory of Silicon Materials School of Materials Science and Engineering Zhejiang University Hangzhou Zhejiang 310027 China; ^5^ Institute of Advanced Semiconductors & Zhejiang Provincial Key Laboratory of Power Semiconductor Materials and Devices ZJU‐Hangzhou Global Scientific and Technological Innovation Center Hangzhou Zhejiang 311200 China; ^6^ State Key Laboratory of Flexible Electronics & Institute of Advanced Materials Nanjing Tech University Nanjing Jiangsu 211816 China; ^7^ Zhejiang Laboratory Hangzhou Zhejiang 311100 China

**Keywords:** ab initio calculations, hybrid organic–inorganic perovskites, molecular dynamics simulations, thermoelectrics

## Abstract

Due to their tantalizing prospect of heat‐electricity interconversion, hybrid organic–inorganic perovskites have sparked considerable research interests recently. Nevertheless, understanding their complex interplay between the macroscopic properties, nonintuitive transport processes, and basic chemical structures still remains far from completion, although it plays a fundamental role in systematic materials development. On the basis of multiscale first‐principles calculations, this understanding is herein advanced by establishing a comprehensive picture consisting of atomic and charge dynamics. It is unveiled that the ultralow room‐temperature lattice thermal conductivity (≈0.20 W m^−1^ K^−1^) of hybrid perovskites is critical to their decent thermoelectric figure of merit (≈0.34), and such phonon‐glass behavior stems from not only the inherent softness but also the strong anharmonicity. It is identified that the 3D electrostatic interaction and hydrogen‐bonded networks between the PbI^3−^ cage and embedded cations result in the strongly coupled motions of inorganic framework and cation, giving rise to their high degree of anharmonicity. Furthermore, such coupled motions bring about low‐frequency optical vibrational modes, which leads to the dominant role of electron scattering with optical phonons in charge transport. It is expected that these new atomistic‐level insights offer a standing point where the performance of thermoelectric perovskites can be further enhanced.

## Graphical abstract

The understanding of thermoelectric transport in hybrid perovskites is advanced by creating an atomistic picture consisting of lattice and charge dynamics. The low lattice thermal conductivity is critical to their decent figure of merit, and the complex interactions between PbI_3_^−^ and cations lead to the coupled motions of inorganic framework and cations, resulting in their strong anharmonicity.

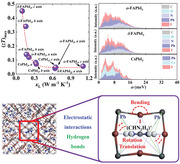

## 1 Introduction

Thermoelectric (TE) materials provide an eco‐friendly solution for directly interconverting thermal and electrical energy. Specifically, a TE generator can turn ubiquitous heat into electrical power based on the Seebeck effect, and electricity can drive a TE material to work as a heat pump for cooling through the Peltier effect.^[^ ^1^ ^]^ The efficiency of a TE material is primarily dictated by a dimensionless figure of merit, *zT* = *S* ^2^ *σT*/(*κ*_e_ + *κ*_L_), where *S*, *σ*, *κ*_e_, and *κ*_L_ are Seebeck coefficient, conductivity, electronic thermal conductivity, and lattice thermal conductivity, respectively, and *S* ^2^ *σ* is defined as power factor.^[^ ^2^ ^]^ A superb TE material must possess a high figure of merit, which calls for simultaneously boosting its Seebeck coefficient and conductivity yet suppressing its thermal conductivity.^[^ ^3^ ^]^ Nevertheless, in practice, the nontrivial interrelationships among such parameters offer an exacting challenge for enhancing its performance. For instance, increasing the conductivity leads to the dramatic attenuation of Seebeck coefficient and concurrently the elevation of electronic thermal conductivity.^[^ ^4^ ^]^ In recent years, researchers have been making unremitting efforts on the development of high‐performance inorganic TE materials, and a variety of effective design strategies have been proposed, such as optimizing the electrical transport properties by band engineering,^[^ ^5^, ^6^ ^]^ inhibiting the thermal transport by involving phonon scattering at various length scales,^[^ ^7^, ^8^ ^]^ and so forth.

Compared with the conventional inorganic TE materials, the burgeoning hybrid organic–inorganic ones exhibit a series of unique characteristics, including flexibility, low cost, and easy processing, which endows them to be applicable for wearable energy harvesting and temperature control.^[^ ^9^, ^10^ ^]^ Importantly, hybrid organic–inorganic composites open up a fascinating opportunity to decouple the electron and phonon transports.^[^ ^11^ ^]^ Furthermore, the Ångström‐level interactions between organic and inorganic components may modulate the geometric structures, electronic properties, and transport behaviors, thus possibly offering paradigm‐changing mechanisms to optimize the performance.^[^ ^12^, ^13^ ^]^

Lately, due to their tantalizing prospect for TE applications, the innovative hybrid organic–inorganic perovskites have sparked extensive research enthusiasm. For example, it is experimentally demonstrated that a novel hybrid perovskites, (4Tm)_2_FASn_2_I_7_ (4Tm = 3‴,4′‐dimethyl[2,2′:5′,2″:5″,2‴‐quaterthiophen]‐5‐yl)ethan‐1‐ammonium, and FA = formamidinium) thin films show a large power factor of 7.07 µW m^−1^ K^−2^ at 343 K with a conductivity of 5.07 S cm^−1^ and a Seebeck coefficient of 118.1 µV K^−1^.^[^ ^14^ ^]^ Moreover, Mettan et al. prove that by doping optimization, the measured figure of merit of hybrid perovskites, CH_3_NH_3_MI_3_ (M = Pb, Sn) single crystals can be enhanced to 0.13 at room temperature.^[^ ^15^ ^]^ It is noteworthy that previous experimental examinations of TE properties in hybrid organic–inorganic perovskites mainly concentrate on the materials preparation and performance characterization. Indubitably, further improving the TE performance of hybrid organic–inorganic perovskites, and systematically designing new materials urgently require not only advancing the basic understanding on the nonintuitive charge and thermal transports, but also establishing the reliable correlation among such processes, properties, and chemical structures. Nonetheless, fundamental investigations of TE response in hybrid organic–inorganic perovskites have been sorely lacking. As a result, their microscopic transport mechanisms still remain poorly understood, which inevitably hinders the further refinement of their performance, and the discovery of new materials.

To overcome the aforementioned challenge, by taking two representative hybrid organic–inorganic perovskites (i.e., *α*‐ and *δ*‐FAPbI_3_) as examples, we conduct ab initio studies to probe their *p*‐type TE properties and the underlying heat‐electricity interconversion mechanisms. Here, a multiscale computational scheme combining first‐principles Born–Oppenheimer molecular dynamics (MD), density functional theory (DFT), density functional perturbation theory, Einstein relationship, electronic and phonon Boltzmann transport theory, Fröhlich polaron model, deformation potential (DP) model, and Brooks–Herring approach is employed. We establish a general atomistic‐level framework to understand the TE response in perovskite materials by correlating their nontrivial transport processes to basic chemical structures.

## 2 Results and Discussion

### 2.1 TE Power Factor and Figure of Merit

**Figure 1:**
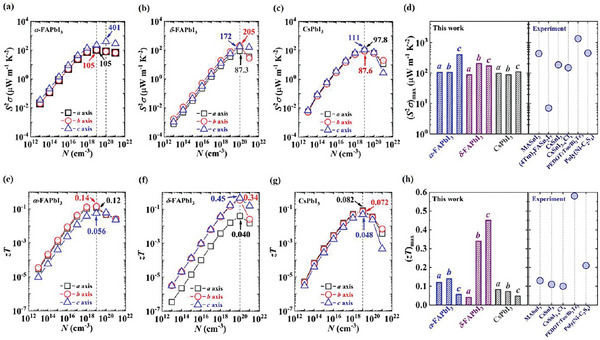
The computed *a*‐ (black square), *b*‐ (red circle), and *c*‐axis (blue triangle) TE power factor (*S* ^2^ *σ*) varying with the hole concentration (*N*) for crystalline a) *α*‐FAPbI_3_, b) *δ*‐FAPbI_3_, and c) CsPbI_3_, respectively, at room temperature. The maximum power factors at the optimal doping levels are displayed in the figure. d) Comparison of the calculated room‐temperature *a*‐, *b*‐, and *c*‐axis maximum power factor [(*S* ^2^ *σ*)_max_] for crystalline *α*‐FAPbI_3_ (blue), *δ*‐FAPbI_3_ (purple), and CsPbI_3_ (gray), and the measured power factor (blue circle) for some state‐of‐the‐art perovskites and hybrid organic–inorganic materials. The computed *a*‐ (black square), *b*‐ (red circle), and *c*‐axis (blue triangle) TE figure of merit (*zT*) varying with the hole concentration (*N*) for crystalline e) *α*‐FAPbI_3_, f) *δ*‐FAPbI_3_, and g) CsPbI_3_, respectively, at room temperature. The maximum figure of merit at the optimal doping level are displayed in the figure. h) Comparison of the calculated room‐temperature *a*‐, *b*‐, and *c*‐axis maximum figure of merit [(*zT*)_max_] for crystalline *α*‐FAPbI_3_ (blue), *δ*‐FAPbI_3_ (purple), and CsPbI_3_ (gray), and the measured figure of merit (blue circle) for some current state‐of‐the‐art perovskites and hybrid organic–inorganic materials. The corresponding references of the published experimental works in (d) and (h) are summarized in Tables S10 and S11 in the Supporting Information, respectively.

To probe the room‐temperature *p*‐type TE properties of our studied materials, we use the Boltzmann transport theory^[^ ^16^ ^]^ to calculate their Seebeck coefficient, conductivity, electronic thermal conductivity, and power factor. We describe the electron–optical phonon, electron–acoustic phonon, and electron–charged impurity interactions by using the Fröhlich polaron model,^[^ ^17^, ^18^ ^]^ DP model,^[^ ^19^ ^]^ and Brooks–Herring approach,^[^ ^20^ ^]^ respectively. The computational details can be found in the Experimental Section and Section S1 of the Supporting Information. To achieve the maximum power factor and figure of merit, and to attain the optimal doping level, we regulate the TE transport coefficients (including Seebeck coefficient, conductivity, and electronic thermal conductivity) of our studied systems by controlling their hole concentration. It is found that as the hole concentration increases, the Seebeck coefficient dramatically drops, while the conductivity and electronic thermal conductivity increase (Figure S11, Supporting Information). Consequently, a peak value of power factor and maximum figure of merit appear at the optimal hole concentration (**Figure** **1**a–c,e–g).

Figure 1a,b exhibits that the maximum power factors of crystalline *α*‐ and *δ*‐FAPbI_3_ fall in the range of 80−400 µW m^−1^ K^−2^ at 10^19^−10^20^ cm^−3^, slightly higher than those of crystalline CsPbI_3_ (80−110 µW m^−1^ K^−2^ at 10^19^ cm^−3^) (Figure 1c). Overall, our predicted power factors of hybrid organic–inorganic perovskites are comparable with the measured results for a series of representative perovskites and hybrid organic–inorganic materials (Figure 1d). For instance, the crystalline *α*‐FAPbI_3_ shows the *c*‐axis optimal power factor of 401 µW m^−1^ K^−2^, comparable with the experimental one (433 µW m^−1^ K^−2^) of the polycrystalline hybrid halide perovskites, CH_3_NH_3_SnI_3_ at room temperature (Figure 1d).^[^ ^15^ ^]^

It is noteworthy that *α*‐FAPbI_3_ single crystal shows the maximum figure of merit of 0.12 and 0.14 along *a*‐ and *b*‐axis directions, respectively, at 10^19^ cm^−3^ (Figure 1e), and crystalline *δ*‐FAPbI_3_ possesses the optimal *b*‐axis figure of merit of 0.34 and *c*‐axis value of 0.45 at the hole concentration of 10^20^ cm^−3^ (Figure 1f). Compared with the hybrid perovskites, the crystalline CsPbI_3_ shows the relatively lower optimal figure of merit, i.e., 0.082, 0.072, and 0.048 along the *a*, *b*, and *c* axes, respectively (Figure 1g). Moreover, our computed maximum figure of merits of the studied systems are consistent with the experimental results for some typical perovskites and hybrid materials (Figure 1h). Specifically, crystalline *α*‐FAPbI_3_ exhibits the optimal figure of merit of 0.12 (*a* axis) and 0.14 (*b* axis), comparable with the measured one (0.13) of the polycrystalline CH_3_NH_3_SnI_3_ under room temperature (Figure 1h).^[^ ^15^ ^]^ These results corroborate that for hybrid organic–inorganic perovskites, continuously regulating their TE transport coefficients (e.g., by intentionally chemical doping) offers an effective route to achieve the enhancement of figure of merit.

### 2.2 Thermal Transport Properties

**Figure 2:**
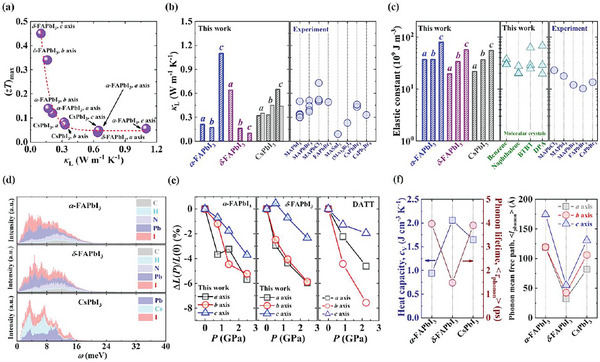
a) The computed *a*‐, *b*‐, and *c*‐axis maximum TE figure of merit [(*zT*)_max_] as a function of the lattice thermal conductivity (*κ*_L_) for crystalline *α*‐FAPbI_3_, *δ*‐FAPbI_3_, and CsPbI_3_ at room temperature. The red dashed line shows the trend. b) Comparison of the calculated room‐temperature *a*‐, *b*‐, and *c*‐axis lattice thermal conductivity (*κ*_L_) for crystalline *α*‐FAPbI_3_ (blue), *δ*‐FAPbI_3_ (purple), and CsPbI_3_ (gray), and the measured ones (blue circle) for some representative perovskites. For crystalline CsPbI_3_, its lattice thermal conductivities obtained by Einstein relationship (gray diagonal columns) and phonon Boltzmann transport theory (gray filled columns) are both present. c) The calculated *a*‐, *b*‐, and *c*‐axis elastic constant for crystalline *α*‐FAPbI_3_ (blue), *δ*‐FAPbI_3_ (purple), and CsPbI_3_ (gray). The computed elastic constant of four typical molecular crystals [including benzene, naphthalene, [1]benzothieno[3,2‐b]benzothiophene (BTBT), and 2,6‐diphenylanthracene (DPA), green triangle], and the measured elastic constant (blue circle) for some representative perovskites are shown for comparison. The corresponding references of the published experimental works in (b) and (c) are summarized in Tables S12 and S13 in the Supporting Information, respectively. d) The vibrational density of states (DOS) for crystalline *α*‐FAPbI_3_, *δ*‐FAPbI_3_, and CsPbI_3_ at room temperature, respectively. The contributions of carbon, hydrogen, nitrogen, lead, iodine, and cesium atoms are displayed in gray, green, light blue, blue, red, and green, respectively. e) The relationships between decreasing ratio of the lattice parameters [Δ*L*(*P*)/*L*(0)] and external pressure (*P*) for crystalline *α*‐FAPbI_3_, *δ*‐FAPbI_3_, and one representative molecular crystal, dianthra[2,3‐*b*:2’,3’‐*f*]thieno[3,2‐*b*]thiophene (DATT). The change ratio of the lattice parameters is defined as Δ*L*(*P*)/*L*(0) = [*L*(*P*) − *L*(0)]/*L*(0), where *L*(*P*) and *L*(0) are the lattice parameters (i.e., *a*, *b*, and *c*) at the pressure *P* and in the ambient condition, respectively. f) The heat capacity per volume at constant volume (*c*_V_, blue square), phonon lifetime (*τ*_phonon_, red circle), and *a*‐ (gray square), *b*‐ (red circle), and *c*‐axis (blue triangle) phonon mean free path (*l*_phonon_) for crystalline *α*‐FAPbI_3_, *δ*‐FAPbI_3_, and CsPbI_3_, respectively, at room temperature.

To uncover the origin of remarkable TE figure of merit of hybrid organic–inorganic perovskites, we delve into their thermal transport properties. On the basis of equilibrium ab initio MD simulations, both the Einstein relationship^[^ ^21^ ^]^ and phonon Boltzmann transport theory^[^ ^22^ ^]^ are herein employed to quantify their lattice thermal conductivity, and the computational details are displayed in the Experimental Section and Section S1 of the Supporting Information. **Figure** **2**a reveals that for our studied materials, their maximum figure of merits exhibit an obviously monotonic decrease trend with the increase of lattice thermal conductivity, which demonstrates that the suppressed thermal transport holds the key to the high‐performance TE response in hybrid perovskites. It is worth noting that these systems show low lattice thermal conductivities (0.1−1 W m^−1^ K^−1^) at room temperature, agreeing with the experimental ones for some representative perovskites (Figure 2b). As an example, the measurements by a steady‐state method prove that the single‐crystal CH_3_NH_3_PbI_3_ possesses a low lattice thermal conductivity of 0.5 W m^−1^ K^−1^ at room temperature (Figure 2b).^[^ ^23^ ^]^ In addition, the lattice thermal conductivities of crystalline CsPbI_3_ computed by Einstein relationship are accordant with those obtained by phonon Boltzmann transport equation (Figure 2b), evidencing the rationality of the ab initio MD methodology for heat transport.

To clarify the thermal transport mechanism of our studied materials, we calculate their elastic constants, and find that they are in the range of 20−80 × 10^9^ J m^−3^ (Figure 2c). Such predicted results are in line with the measured ones for some typical perovskite materials; for instance, the experiments demonstrate that the single‐crystal CH_3_NH_3_PbI_3_ possesses an elastic constant of 23 × 10^9^ J m^−3^ by using a nanoindentation technique (Figure 2c).^[^ ^24^ ^]^ Moreover, it is known that, for crystalline organic materials, the isolated molecules are loosely assembled primarily by the van der Waals forces, and such weak intermolecular interactions are the root of their small elastic constants.^[^ ^25^ ^]^ Therefore, to compare the elastic constants of perovskites with those of inherently soft molecular solids, we compute the elastic constants and phonon distributions for four representative organic molecular crystals, including benzene, naphthalene, BTBT, and DPA (Figure 2c and Figure S12, Supporting Information). Figure 2c exhibits that the elastic constants of these molecular crystals fall in the range of 20−70 × 10^9^ J m^−3^, comparable with those of perovskites, apparently showing the inherent softness for perovskite materials. A small elastic constant indicates a low group velocity of longitudinal acoustic mode (2 × 10^3^−4 × 10^3^ m s^−1^) (Table S14, Supporting Information), thereby inhibiting the thermal transport. Thus, we can conclude that the soft nature of perovskite crystals is responsible for their low lattice thermal conductivity.

The frequency calculation for internal vibrations of isolated organic cation, (CHN_2_H_4_)^+^ demonstrates that such vibrational modes are located at relatively high‐frequency region (500−3700 cm^−1^, i.e., 62−459 meV) (Table S15, Supporting Information). However, the simulated vibrational DOS shows that except the internal vibrations of organic cation, all the vibrational modes are located at the low‐frequency region (0−24 meV) (Figure 2d and Figure S13, Supporting Information). Furthermore, for *α*‐FAPbI_3_, *δ*‐FAPbI_3_, and CsPbI_3_ single crystals, each element contributes to the low‐energy vibrations (Figure 2d), proving that both the anion framework and embedded cation participate in the low‐frequency vibrational modes. Hence, the low‐frequency distributed vibrations also suggest the soft nature of perovskite crystals.

To further verify the inherent softness of crystalline perovskites, the external hydrostatic pressure effect on the crystallographic structures of *α*‐ and *δ*‐FAPbI_3_ is modeled (Figure 2e and Figure S14, Supporting Information). It is found that from ambient pressure to 2.5 GPa, the decreasing ratios of the lattice parameters along the *a*, *b*, and *c* axes are 5.69%, 5.23%, and 3.71% for *α*‐FAPbI_3_, and 5.86%, 5.86%, and 2.33% for *δ*‐FAPbI_3_, respectively, comparable with the calculated results (4.61%, 7.56%, and 1.95% along the *a*, *b*, and *c* axes) for molecular crystal, DATT (Figure 2e). This result further confirms the soft nature of crystalline perovskites.

By analyzing their heat capacity, phonon lifetime, and phonon mean free path, we reveal that the crystalline perovskites not only exhibit the inherent softness, but also possess strong phonon–phonon interactions. Figure 2f displays that the heat capacities of crystalline *α*‐FAPbI_3_, *δ*‐FAPbI_3_, and CsPbI_3_ are around 1−2 J cm^−3^ K^−1^. More importantly, they show short phonon lifetime (1−4 ps) and small phonon mean free path (30−180 Å) at room temperature (Figure 2f), clearly demonstrating the strong anharmonic interactions between phonons. Such strong vibrational anharmonicity can be attributed to the low‐energy phonon populations (0−24 meV), particularly high‐density low‐frequency optical modes, as shown in our computed vibrational DOS (Figure 2d) and phonon dispersion relations (Figure S9, Supporting Information). Low‐energy optical phonons in our studied materials inevitably increase the scattering probability of acoustic phonons, yielding their high degree of anharmonicity, short phonon lifetime, and ultralow lattice thermal conductivity. In addition, our predicted phonon lifetime and phonon mean free path agree with the reported experimental ones (picosecond phonon lifetime and nanometer mean free path) for hybrid perovskite CH_3_NH_3_PbI_3_ single crystals based on the inelastic neutron spectroscopy.^[^ ^26^ ^]^

### 2.3 Atomistic‐Level Origin of Anharmonicity

**Figure 3:**
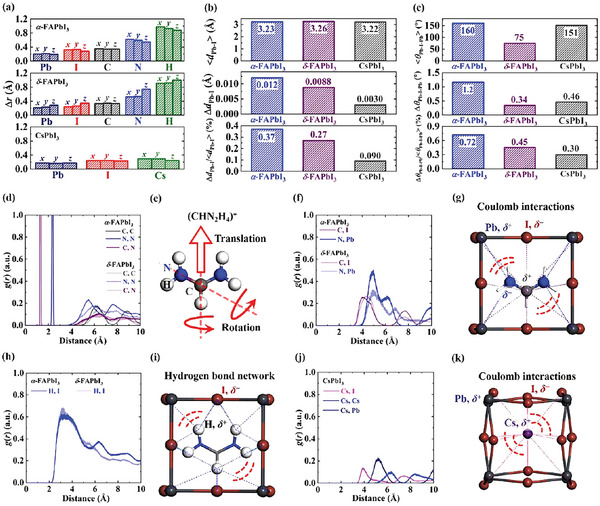
a) The standard deviation of lead (dark blue), iodine (red), carbon (gray), nitrogen (blue), hydrogen (green), and cesium (green) atom position (Δ ***r***) for crystalline *α*‐FAPbI_3_, *δ*‐FAPbI_3_, and CsPbI_3_, respectively, at room temperature. The atomic position evolution with time is displayed in Figure S15 in the Supporting Information. b) The average (〈*d*_Pb − I_〉) and standard deviation (Δ*d*_Pb − I_) of Pb—I bond length for crystalline *α*‐FAPbI_3_ (blue), *δ*‐FAPbI_3_ (purple), and CsPbI_3_ (gray), respectively, at room temperature. The ratio between the standard deviation of Pb—I bond length and the average one (Δ*d*_Pb − I_/〈*d*_Pb − I_〉) for crystalline *α*‐FAPbI_3_ (blue), *δ*‐FAPbI_3_ (purple), and CsPbI_3_ (gray), respectively, at room temperature. c) The average (〈*θ*_Pb − I − Pb_〉) and standard deviation (Δ*θ*_Pb − I − Pb_) of Pb‐I‐Pb bond angle for crystalline *α*‐FAPbI_3_ (blue), *δ*‐FAPbI_3_ (purple), and CsPbI_3_ (gray), respectively, at room temperature. The ratio between the standard deviation of Pb—I—Pb bond angle and the average one (Δ*θ*_Pb − I − Pb_/〈*θ*_Pb − I − Pb_〉) for crystalline *α*‐FAPbI_3_ (blue), *δ*‐FAPbI_3_ (purple), and CsPbI_3_ (gray), respectively, at room temperature. d) The radial distribution function [*g*(*r*)] of the C∙∙∙C, N∙∙∙N, and C∙∙∙N for crystalline *α*‐ and *δ*‐FAPbI_3_ at room temperature. e) The geometry of the (CHN_2_H_4_)^+^ and its thermal motion modes. f) The radial distribution function [*g*(*r*)] of the C∙∙∙I and N∙∙∙Pb for crystalline *α*‐ and *δ*‐FAPbI_3_ at room temperature. g) The Coulomb interaction network of N∙∙∙Pb and C∙∙∙I for crystalline *α*‐FAPbI_3_. h) The radial distribution function [*g*(*r*)] of the H∙∙∙I for crystalline *α*‐ and *δ*‐FAPbI_3_ at room temperature. i) The hydrogen bond network of H∙∙∙I for crystalline *α*‐FAPbI_3_. j) The radial distribution function [*g*(*r*)] of the Cs∙∙∙I, Cs∙∙∙Cs, and Cs∙∙∙Pb for crystalline CsPbI_3_ at room temperature. k) The Coulomb interaction network of Cs∙∙∙I for crystalline CsPbI_3_. The lead, iodine, carbon, nitrogen, hydrogen, and cesium atoms are shown in dark gray, brown, gray, blue, white, and purple, respectively.

To elucidate the nonintuitive correlation between the strong anharmonic interactions and fundamental chemical structure of our studied systems, we further explore their atomistic‐level structural dynamics at room temperature by carefully analyzing ab initio MD trajectories. **Figure** **3**a and Table S16 in the Supporting Information exhibit the atomic position standard deviation of each element, and it is demonstrated that for crystalline *α*‐ and *δ*‐FAPbI_3_, the C, N, and H atoms in organic cation, (CHN_2_H_4_)^+^ show larger standard deviation of atom position (0.3−1 Å) than Pb and I atoms (0.1−0.3 Å) in PbI^3−^ sublattice. However, for CsPbI_3_ single crystal, the Pb, I, and Cs have similar atomic position standard deviation (0.1−0.3 Å) (Figure 3a and Table S16, Supporting Information). This phenomenon reveals that compared with the inorganic perovskites, the organic cations in hybrid ones possess larger thermally induced motion.

The thermally induced geometric fluctuation for PbI^3−^ sublattice is further quantitatively investigated (Figure 3b,c and Figure S16 and Table S17, Supporting Information). Figure 3b and Table S17 in the Supporting Information show that the room‐temperature standard deviation of Pb—I bond length (0.003−0.012 Å), and the ratio between its standard deviation and average bond length (0.09−0.37%) are small for crystalline *α*‐FAPbI_3_, *δ*‐FAPbI_3_, and CsPbI_3_, despite the thermally induced atomic motion. Nevertheless, the standard deviation of Pb—I—Pb bond angle (0.34°−1.2°), and the ratio between its standard deviation and average bond angle (0.30−0.72%) are relatively larger (Figure 3c and Table S17, Supporting Information). These results prove that for the inorganic frameworks of crystalline perovskites, the thermally induced Pb—I—Pb bond angle change (i.e., bending vibration) is much more significant than Pb—I bond length change (i.e., stretching vibration).

To uncover the thermal motion of cations, and the interplay between it and PbI^3−^ sublattice, the room‐temperature radial distribution functions of our studied systems are probed (Figure 3d–k and Figure S17, Supporting Information). The shark peaks of N∙∙∙N (at ≈2.33 Å) and C—N (at ≈1.32 Å) indicate the unobvious intramolecular vibrations in (CHN_2_H_4_)^+^ for crystalline *α*‐ and *δ*‐FAPbI_3_ (Figure 3d), in line with the aforementioned computational results of vibrational frequency for isolated organic cation. Whereas, the distributions of C∙∙∙C, N∙∙∙N, and C∙∙∙N at distances of larger than 4 Å markedly broaden (Figure 3d), suggesting the significant rotational and translational modes of (CHN_2_H_4_)^+^ in crystalline *α*‐ and *δ*‐FAPbI_3_ at room temperature (Figure 3e). Interestingly, the similar results are also confirmed in the CH_3_NH_3_PbI_3_ single crystal based on the high‐resolution time‐of‐flight quasi‐elastic and inelastic neutron scattering measurements.^[^ ^27^ ^]^ Besides, we notice that the theoretical exploration based on classical MD simulations proves that the rotational motion of methylammonium cations in crystalline CH_3_NH_3_PbI_3_ shows an important suppression effect in thermal transport.^[^ ^28^ ^]^ Our present findings for *α*‐ and *δ*‐FAPbI_3_ based on first‐principles MD simulations are consistent with such results.

Furthermore, our atomic charge analysis demonstrates that for crystalline *α*‐ and *δ*‐FAPbI_3_, the lead atoms in PbI^3−^ sublattice are positively charged, while the iodine atoms are negatively charged (Table S18, Supporting Information). In organic cations, the carbon and hydrogen atoms are positively charged, yet the nitrogen atoms are negatively charged (Table S18, Supporting Information). Such characteristic naturally results in Coulomb interaction and hydrogen bond networks between the inorganic frameworks and organic cations, and thereby giving rise to the coupled motions of PbI^3−^ cage and (CHN_2_H_4_)^+^.

The computed room‐temperature radial distribution functions of the C∙∙∙I, N∙∙∙Pb (Figure 3f,g), and H∙∙∙I (Figure 3h,i) for crystalline *α*‐ and *δ*‐FAPbI_3_ further corroborate the aforementioned statement. Specifically, it is observed that the coupled motions between the PbI^3−^ cage and (CHN_2_H_4_)^+^ induced by the electrostatic interaction networks markedly broaden the distance distributions of C∙∙∙I and N∙∙∙Pb, compared with those for the ideal crystals (Figure 3f,g and Figure S18, Supporting Information). Likewise, the distance distributions of H∙∙∙I is broadened by the coupled motions between the PbI^3−^ cage and (CHN_2_H_4_)^+^ caused by the hydrogen bond networks (Figure 3h,i). As a result, the complex interacting net (including Coulomb force and hydrogen bond) in *α*‐ and *δ*‐FAPbI_3_ single crystals brings about the motions of inorganic framework coupled strongly to the cation motions [specifically, Pb—I—Pb bending vibrations, and rotational and translational modes of (CHN_2_H_4_)^+^].

Additionally, Figure 3j,k shows that the coupled motions between the Cs^+^ and PbI^3−^ cage induced by the Coulomb interactions also broaden the distance distributions of Cs∙∙∙I and Cs∙∙∙Pb in crystalline CsPbI_3_ at room temperature, which further evidences the aforementioned conclusions for *α*‐ and *δ*‐FAPbI_3_ single crystals.

### 2.4 Charge Transport from the Standpoint of Electronic Structure and Lattice Dynamics

**Figure 4:**
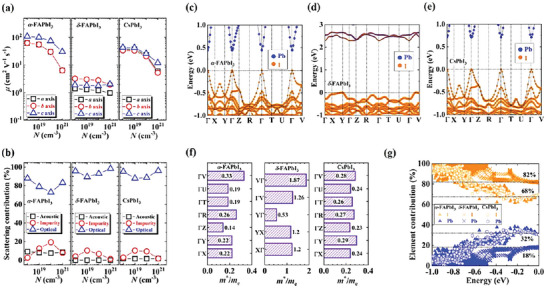
a) The computed *a*‐ (black square), *b*‐ (red circle), and *c*‐axis (blue triangle) hole mobility (*μ*) varying with the concentration (*N*) for crystalline *α*‐FAPbI_3_, *δ*‐FAPbI_3_, and CsPbI_3_, respectively, at room temperature. b) The scattering contribution of acoustic phonon (black square), charged impurity (red circle), and optical phonon (blue triangle) varying with the hole concentration (*N*) for crystalline *α*‐FAPbI_3_, *δ*‐FAPbI_3_, and CsPbI_3_, respectively, at room temperature. Here, the charge carrier scattering contribution is defined as $\left(1/\langle \tau_{i}\rangle \right)/\left(\sum_{i}1/\langle \tau_{i}\rangle \right)\times 100\%$, where 〈*τ*_*i*_〉 is the average hole scattering time caused by acoustic phonon, charged impurity, or optical phonon. The detailed data for average hole scattering times contributed from acoustic phonon, charged impurity, and optical phonon are summarized in Tables S23−S26 in the Supporting Information. The element projected band structures for crystalline c) *α*‐FAPbI_3_, d) *δ*‐FAPbI_3_, and e) CsPbI_3_, respectively. The symbol size denotes the relative weight of the component ratio. The reciprocal coordinates of high‐symmetry *k*‐points in the first Brillouin zone are **Γ** = (0, 0, 0), **X** = (0.5, 0, 0), **Y** = (0, 0.5, 0), **Z** = (0, 0, 0.5), **R** = (0.5, 0.5, 0.5), **T** = (0, 0.5, 0.5), **U** = (0.5, 0, 0.5), and **V** = (0.5, 0.5, 0), respectively. The lead and iodine atoms are shown in blue and orange circles, respectively. The nonprojected band structures are displayed in the gray lines. The computed band structures for *α*‐FAPbI_3_, *δ*‐FAPbI_3_, and CsPbI_3_ single crystals with and without spin–orbital coupling are represented in Figure S21 in the Supporting Information. f) The calculated hole effective mass (m*/*m*_e_) at band edge for crystalline *α*‐FAPbI_3_, *δ*‐FAPbI_3_, and CsPbI_3_, respectively. The detailed data for their hole effective masses are summarized in Table S27 in the Supporting Information. g) The contribution of iodine (orange) and lead (blue) elements to the valence bands varying with the band energy for crystalline *α*‐FAPbI_3_ (triangle), *δ*‐FAPbI_3_ (cross), and CsPbI_3_ (circle), respectively. The valence band edge is at the position where the energy is zero.

In addition to the above discussed thermal transport, charge dynamics is also a key process in the TE response of a solid‐state material.^[^ ^29^ ^]^ Hence, to shed light on the nonintuitive charge transport mechanism of our studied materials, we extract their room‐temperature hole mobilities. **Figure** **4**a exhibits the dependence of hole mobilities on the concentration of *α*‐FAPbI_3_, *δ*‐FAPbI_3_, and CsPbI_3_ single crystals, and it is demonstrated that their hole mobilities tend to decrease with the increase of concentration. Importantly, the hole mobilities of crystalline *δ*‐FAPbI_3_ (0.965−3.21 cm^2^ V^−1^ s^−1^) are much smaller than those of crystalline *α*‐FAPbI_3_ (6.31−107 cm^2^ V^−1^ s^−1^) and CsPbI_3_ (5.23−42.8 cm^2^ V^−1^ s^−1^) (Figure 3a), demonstrating the superior charge transport properties for the latter two systems, and the reason for such phenomena will be expounded below. Besides, our calculated results show that the crystalline *α*‐FAPbI_3_ possesses the room‐temperature hole mobilities of 6.31 cm^2^ V^−1^ s^−1^ along the *a* and *b* axes at the concentration of 10^21^ cm^−3^ (Figure 4a), agreeing with the measured mobility (4.4 cm^2^ V^−1^ s^−1^) for FAPbI_3_ single crystal,^[^ ^30^ ^]^ which proves the reliability of our computational method.

It is known that for crystalline materials, their charge transport properties are inherently dictated not only by the electronic structures but also by the various electron scattering processes.^[^ ^31^, ^32^ ^]^ Therefore, by comprehensively analyzing the contributions of different electron scattering processes, the microscopic charge transport mechanism of crystalline *α*‐FAPbI_3_, *δ*‐FAPbI_3_, and CsPbI_3_ is explored. The dependence of scattering contribution on the hole concentration in Figure 4b shows that for our studied systems, the optical phonon scattering contributes to ≈70−90%, yet the contributions of charged impurity scattering and acoustic phonon scattering are ≤20% and ≤10%, respectively, evidencing the governing role of optical phonon scattering in the charge transport properties at room temperature. Additionally, by probing the contributions of different electron scattering processes (including the acoustic phonon, charged impurity, and optical phonon scattering) to the computed room‐temperature conductivity, power factor, electronic thermal conductivity, and mobility for *α*‐FAPbI_3_, *δ*‐FAPbI_3_, and CsPbI_3_ single crystals (Figure S19 and Tables S19−S22, Supporting Information), the dominant role of optical phonon scattering in the charge and TE transport properties are further corroborated.

From the point of view of electronic structures, the calculated band structures for *α*‐FAPbI_3_, *δ*‐FAPbI_3_, and CsPbI_3_ single crystals reveal that their valence bands are determined mainly by the iodine atoms in the PbI^3−^ sublattice (Figure 4c–e), consistent with their charge density isosurfaces (Figure S20, Supporting Information). Furthermore, our aforementioned conclusion states that the complex interactions (especially electrostatic interactions) between the PbI^3−^ cage and embedded cations leads to the large‐amplitude vibrations (especially Pb—I—Pb bending vibrations with low frequency and large atomic displacement) (Figure 3). In addition, our atomic charge analysis shows that in the polar PbI^3−^ frameworks, the lead and iodine atoms are positively and negatively charged, respectively (Table S18, Supporting Information), which can further intensify the motions of PbI^3−^ sublattice. Importantly, a direct consequence of Pb—I—Pb bending vibrations coupled by the rotational and translational modes of embedded cations is to introduce the low‐frequency large‐amplitude polar optical modes, as shown in our simulated vibrational DOS (Figure 2d). Thus, in perovskite materials, such optical phonons have great impact on the electronic structures, especially the valence bands controlled mainly by the iodine atoms, and accordingly, the charge and TE transport are limited by the electron scattering with optical phonons. Incidentally, on the basis of time‐dependent DFT and nonadiabatic MD, it is reported that the motions of the inorganic and organic components of the hybrid perovskite also play a key role in the nonradiative electron–hole recombination and excited‐state lifetime.^[^ ^33^ ^]^

To figure out the underlying reason for the superior mobilities of crystalline *α*‐FAPbI_3_ and CsPbI_3_, the hole effective masses of valence band edges are extracted. Figure 4f shows that the evaluated hole effective masses of single‐crystal *α*‐FAPbI_3_ (0.14−0.33*m*_e_) and CsPbI_3_ (0.23−0.29*m*_e_) are much lighter than those of *δ*‐FAPbI_3_ (0.53−1.87*m*_e_), demonstrating more delocalized charge carrier for the former two systems. This result is also confirmed by the calculated band structures (Figure 4c–e); specifically, *α*‐FAPbI_3_ and CsPbI_3_ single crystals have much more dispersive valence bands (≈800 meV) than *δ*‐FAPbI_3_ (≈400 meV) (Figure 4c–e). Generally, a large band dispersion leads to a small probability that the charge carrier with a certain energy is scattered to another similar energy state, consequently bringing about a long relaxation time, a long mean free path, and a large mobility.^[^ ^34^ ^]^ More importantly, according to the definition of dimensionless coupling parameter in the Fröhlich polaron model (Equation (S8), Supporting Information), a small effective mass is responsible for a weak electron–optical phonon coupling. Since the charge transport properties of our studied materials are governed by the optical phonon scattering, crystalline *α*‐FAPbI_3_ and CsPbI_3_ with smaller hole effective masses possess weaker electron–optical phonon coupling, and thereby higher mobilities than *δ*‐FAPbI_3_.

To understand the essential cause of the weaker electron–optical phonon coupling for crystalline *α*‐FAPbI_3_ and CsPbI_3_ from the viewpoint of basic chemical structure, the deeper quantitative analysis of contributions of lead and iodine atoms in PbI^3−^ sublattice to their valence bands is performed. Figure 4g demonstrates that for *α*‐FAPbI_3_ and CsPbI_3_ single crystals, the iodine and lead atoms contribute ≈68% and 32%, respectively, to their valence bands, while for *δ*‐FAPbI_3_, the contributions of iodine and lead atoms to its valence bands are around 82% and 18%, respectively. Compared with the *δ*‐FAPbI_3_ single crystal, the crystalline *α*‐FAPbI_3_ and CsPbI_3_ show a smaller difference in the contributions of these two elements to their valence bands. The smaller difference in the contributions of iodine and lead atoms to the valence bands means that the hole is more delocalized in the PbI^3−^ sublattice. Thus, compared with crystalline *δ*‐FAPbI_3_, *α*‐FAPbI_3_ and CsPbI_3_ crystals exhibit more delocalized hole behavior, consequently showing smaller hole effective masses (Figure 4f). A light hole effective mass gives rise to a weak hole–optical phonon coupling. As a result, crystalline *α*‐FAPbI_3_ and CsPbI_3_ possess the superior charge transport properties compared with the *δ*‐FAPbI_3_.

## 3 Conclusion

In summary, understanding the origin of high‐performance TE response in hybrid perovskites has proven to be a long‐standing conundrum, owing to their elusive thermal and charge transport, and unusual crystallographic structures. We herein provide a new atomistic‐level insight to solve such problem by using a multiscale ab initio computational scheme. Our results unveil that the low lattice thermal conductivity holds the key to their decent TE performance for these materials, and more in‐depth studies highlight that not only the inherent softness but also the high degree of anharmonicity is responsible for their phonon‐glass nature. From the perspective of chemical structure, the complex interacting net, including electrostatic interactions and hydrogen bonds, between the PbI^3−^ cage and embedded cations results in the motions of inorganic framework coupled strongly to the cation, thus giving rise to their strong anharmonicity. Furthermore, the leading role of electron scattering with low‐energy optical phonons in charge transport also stems from such interaction network. More broadly, we suggest a general framework to take advantage of engineering nonbonding interactions to achieve high‐efficiency TE perovskites, and anticipate that it will inspire development of this class of materials with increased TE performance.

## 4 Experimental Section

### Einstein Relationship for Lattice Thermal Conductivity based on Equilibrium Ab Initio MD Simulations

The crystallographic data for *α*‐FAPbI_3_, *δ*‐FAPbI_3_, and CsPbI_3_ were attained from the recently published experimental investigations,^[^ ^30^, ^35^ ^]^ and the details for model setup are exhibited in Section S1 of the Supporting Information. Herein, combining equilibrium first‐principles Born–Oppenheimer MD simulations, the Einstein relationship^[^ ^21^ ^]^ was used to evaluate the thermal transport properties at room temperature. In this scheme, the lattice thermal conductivity could be expressed as the diffusion of the energy momentum (***R***)

(1) $$\kappa_{L\;\alpha \beta }=\frac{1}{Vk_{B}T^{2}}\;\lim_{t\to \infty }\frac{1}{2t}\langle \left[R_{\alpha }\left(t\right)-R_{\alpha }\left(0\right)\right]\left[R_{\beta }\left(t\right)-R_{\beta }\left(0\right)\right]\rangle$$

where *V* is the volume; *T* is the temperature; *α* and *β* are the cartesian directions. The energy momentum is defined as $R\;\left(t\right)=\sum_{l}r_{l}\left(t\right)\int_{0}^{t}F_{l}\left(t\right)\cdot v_{l}\left(t\right)dt\;$, where ***r**_l_* (*t*), ***F**_l_* (*t*), and ***v**_l_* (*t*) are the position of the *l*‐th atom, force on the *l*‐th atom, and its velocity at time *t*, respectively. It is worth noting that all the anharmonic interactions were naturally included in this approach due to the anharmonic energy surface obtained from the MD trajectory.^[^ ^36^ ^]^ The detailed methodologies can be found in Section S1 of the Supporting Information.

The ab initio MD simulations were performed by the projector augmented‐wave (PAW) method^[^ ^37^ ^]^ with the Perdew–Burke–Ernzerhof (PBE) exchange‐correlation functional^[^ ^38^ ^]^ including the Grimme's D3 dispersion correction^[^ ^39^ ^]^ in Vienna Ab initio Simulation Package (VASP, version 6.2.1).^[^ ^40^ ^]^ The studied materials were equilibrated in the canonical (NVT) ensemble with Nosé–Hoover thermostat^[^ ^41^ ^]^ for 20 ps at room temperature. The cutoff energy for the plane‐wave basis set was 300 eV, and the energy convergence accuracy was 10^−4^ eV. The supercells of 2 × 2 × 2 (for *α*‐FAPbI_3_), 2 × 2 × 2 (for *δ*‐FAPbI_3_), and 3 × 3 × 2 (for CsPbI_3_) were used, and a *k*‐mesh of 1 × 1 × 1 was utilized. The timestep was set to be 1 fs. By analyzing the MD trajectories, the vibrational DOS, heat capacity, phonon lifetime, and phonon mean free path were simultaneously achieved. The related computational details are summarized in Section S1 of the Supporting Information.

### Phonon Boltzmann Transport Theory for Lattice Thermal Conductivity based on Equilibrium Ab Initio MD Simulations

To corroborate the rationality of the thermal transport properties calculated by the Einstein relationship, the crystalline CsPbI_3_ was taken as an example to compute its room‐temperature lattice thermal conductivity by employing the phonon Boltzmann transport equation. Both the harmonic and third‐order force constants were simultaneously extracted from the ab initio MD trajectories, so the temperature‐caused anharmonic effect on the lattice dynamics was incorporated.^[^ ^22^, ^42^ ^]^ In addition, the phonon distributions, phonon lifetime, and phonon mean free path were attained. The detailed methodologies and computational scheme are shown in Section S1 of the Supporting Information.

### Electronic Structure Characterization

On the basis of the optimized crystallographic structures for *α*‐FAPbI_3_, *δ*‐FAPbI_3_, and CsPbI_3_, the electronic structure calculations were carried out by the PAW method^[^ ^37^ ^]^ with the PBE functional^[^ ^38^ ^]^ in VASP.^[^ ^40^ ^]^ The convergence criterion of the total energy was 10^−5^ eV in the self‐consistent field iteration, and the cutoff energy was 600 eV. The atomic charges were computed by the Bader charge analysis.^[^ ^43^ ^]^ To check the effect of spin–orbital coupling on the valence band structure for the studied systems, their band structures were also calculated by considering such effect. The computational details for geometric structural optimizations are present in Section S1 of the Supporting Information.

### Modeling Hydrostatic Pressure Effect

To explore the hydrostatic pressure effect on the crystalline structures of organic–inorganic hybrid perovskites, the crystal structures were predicted under different external pressures. On the basis of the optimized crystal structures of *α*‐ and *δ*‐FAPbI_3_ under ambient pressure, the unit cell was compressed by scaling the lattice parameters of *a*, *b*, and *c* in a series of random proportion. Then, their atomic positions and lattice constants were relaxed. The values of pressure were obtained by the single point energy calculations, and the pressure of the optimized initial structure was set to be 0 GPa. All the structural optimizations were conducted by using the PAW method^[^ ^37^ ^]^ with the PBE functional^[^ ^38^ ^]^ including the Grimme's D3 dispersion correction^[^ ^39^ ^]^ in VASP.^[^ ^40^ ^]^ The convergence criterion of the total energy was 10^−5^ eV, and the force on each atom was smaller than 0.01 eV Å^−1^. The cutoff energy for the plane‐wave basis set was 600 eV, and the cutoff radius for pair interactions was 50 Å.

### Fröhlich Polaron Model for Electron–Optical Phonon Interactions

Fröhlich polaron model was employed to evaluate the hole relaxation time caused by electron–optical phonon interactions. By considering that the charge carrier in a polar medium was coupled to harmonic optical phonon modes, Feynman nonperturbatively solved the Fröhlich Hamiltonian, and provided the relaxation time expression^[^ ^17^, ^18^ ^]^

(2) $$\tau \;=\frac{3\sqrt{\pi }}{\omega_{\mathrm{eff}}\alpha_{e-\mathrm{ph}}}\;\frac{\sinh\left(\beta /2\right)}{\beta^{5/2}}\frac{w^{3}}{\nu^{3}}\frac{1}{K}$$

where $K\;\left(a,b\right)=\int_{0}^{\infty }\frac{\cos(u)}{{\left[u^{2}+a^{2}-b\cos\left(vu\right)\right]}^{3/2}}du\;$, $a^{2}={\left(\frac{\beta }{2}\right)}^{2}\;+\left(\frac{v^{2}-w^{2}}{w^{2}v}\right)\beta \coth\left(\frac{\beta v}{2}\right)$, and $b\;=\left(\frac{v^{2}-w^{2}}{w^{2}v}\right)\;\frac{\beta }{\sinh(\frac{\beta v}{2})}$. Here, *α*_e − ph_ is the dimensionless Fröhlich parameter quantifying the strength of electron–optical phonon coupling; *ω*_eff_ is the effective frequency of longitudinal optical phonon; $\sinh\left(x\right)\;=\frac{\exp(x)-\exp(-x)}{2}\;$ and $\coth\left(x\right)\;=\frac{\exp(x)+\exp(-x)}{\exp(x)-\exp(-x)}\;$; *w* and *v* are two parameters. To determine these two parameters, the Ōsaka's variational strategy^[^ ^44^, ^45^ ^]^ was utilized to solve the Feynman's model. In Ōsaka's solution, the finite‐temperature free energy of polaron (*F*) with *α*_e − ph_ under the phonon occupation factor *β* = ℏ*ω*_eff_/(*k*_B_ *T*) could be written as *F* = − (*A* + *B* + *C*). Here, $A\;=\frac{3}{\beta }\;\left[\ln\left(\frac{v}{w}\right)-\frac{\ln(2\pi \beta )}{2}-\ln\frac{\sinh(\frac{v\beta }{2})}{\sinh(\frac{w\beta }{2})}\right]$, $B\;=\frac{\alpha v}{\sqrt{\pi }\left[\exp(\beta )-1\right]}\;\int_{0}^{\frac{\beta }{2}}\frac{\exp(\beta -x)+\exp(x)}{\sqrt{w^{2}x\left(1-\frac{x}{\beta }\right)+\frac{Y\left(x\right)\left(v^{2}-w^{2}\right)}{v}}}dx$, $Y\;\left(x\right)=\frac{1}{1-\exp(-v\beta )}\;\left\{1+\exp(-v\beta )-\exp(-vx)-\exp[v(x-\beta )]\right\}$, $C\;=\frac{3(v^{2}-w^{2})}{4v}\;\left[\coth\left(\frac{v\beta }{2}\right)-\frac{2}{v\beta }\right]$. Thus, the variational parameters *w* and *v* could be numerically achieved at room temperature by minimizing the polaron free energy. Furthermore, both the *α*_e − ph_ and *ω*_eff_ could be evaluated at the first‐principles level, and the computational details are displayed in Section S1 of the Supporting Information.

### Deformation Potential (DP) Model for Electron–Acoustic Phonon Interactions

The DP model^[^ ^46^ ^]^ was utilized to quantify the electron–acoustic phonon interactions. In this model, the relaxation time (*τ*_***k***_) had the form

(3) $$\frac{1}{\tau_{k}}=\frac{2\pi }{\hbar \Omega }\;\frac{k_{B}TE_{1}^{2}}{C_{ii}}\sum_{k^{\prime }}\delta \left(E_{k}-E_{k^{\prime }}\right)\left(1-\cos\theta \right)$$

in which *E*_1_ is the DP constant; *C_ii_* (*ii* = *aa*, *bb*, and *cc*) is the elastic constant; *δ*(*E*_***k***_ − *E*_*k*′_) is the Dirac *δ*‐function; *E*_***k***_ is the band energy, and *θ* is the scattering angle. Both the DP constant and elastic constant were achieved from first‐principles calculations, and the computational details can be found in Section S1 of the Supporting Information.

### Brooks–Herring Approach for Electron–Ionized Impurity Interactions

The Brooks–Herring approach^[^ ^20^, ^47^ ^]^ was employed to describe the electron–charged impurity interactions. In this mode, the relaxation time had the form

(4) $$\frac{1}{\tau_{k}}=\frac{2\pi }{\hbar \Omega^{2}}\;\frac{n_{I}{\left(q_{I}e\right)}^{2}}{{\left(\varepsilon_{r}\varepsilon \right)}^{2}}\sum_{k^{\prime }}\frac{1}{{\left(L_{D}^{-2}+{\left|k^{\prime }-k\right|}^{2}\right)}^{2}}\delta \left(E_{k}-E_{k^{\prime }}\right)\left(1-\cos\theta \right)$$

where *n*_I_ is the number of impurities in each unit cell; *q*_I_ is the impurity charge; $L_{D}=\sqrt{\frac{\varepsilon_{r}\varepsilon_{0}k_{B}T}{e^{2}N}}\;$ is the screening length; *N* is the carrier concentration, and *ε*_r_ is the relative permittivity. Assuming the different scattering processes are independent, the total relaxation time could be calculated based on the Matthiessen's rule, namely, $\frac{1}{\tau_{k}}=\sum_{i}\frac{1}{\tau_{i\;k}}$.

### Boltzmann Transport Theory for Conductivity, Seebeck Coefficient, Electronic Thermal Conductivity, and Mobility

On the basis of Boltzmann transport theory,^[^ ^16^, ^48^ ^]^ the conductivity (*σ*), Seebeck coefficient (*S*), and electronic thermal conductivity (*κ*_e_) could be expressed as

(5) $$\sigma \;=\frac{e^{2}}{\Omega }\;\sum_{k}\left[-\frac{\partial f_{0}\left(E_{k},E_{F},T\right)}{\partial E_{k}}\right]v_{k}v_{k}\tau_{k}$$

(6) $$S\;=\frac{e}{\Omega \sigma T}\;\sum_{k}\left[-\frac{\partial f_{0}\left(E_{k},E_{F},T\right)}{\partial E_{k}}\right]\left(E_{k}-E_{F}\right)v_{k}v_{k}\tau_{k}$$

(7) $$\kappa_{0}=\frac{1}{\Omega T}\;\sum_{k}\left[-\frac{\partial f_{0}\left(E_{k},E_{F},T\right)}{\partial E_{k}}\right]{\left(E_{k}-E_{F}\right)}^{2}v_{k}v_{k}\tau_{k}$$

(8) $$\kappa_{e}=\kappa_{0}\;-S^{2}\sigma T$$

where $f_{0}\;\left(E_{k},E_{F},T\right)=\frac{1}{\exp[\left(E_{k}-E_{F}\right)/\left(k_{B}T\right)]+1}\;$ is the Fermi–Dirac distribution function; $v_{k}=\frac{1}{\hbar }\;\nabla_{k}E$ is the group velocity that can be obtained from the density‐functional band structure calculations. The mobility (*μ*) is computed based on the formula, $\mu \;=\frac{\sigma }{eN}\;$. The rigid band approximation was employed and the doping was simulated via shifting the Fermi level; thus, the aforementioned TE transport parameters could be calculated at each Fermi level.

## Conflict of Interest

The authors declare no conflict of interest.

## Supporting information

Supporting Information

## Data Availability

The data that support the findings of this study are available in the supplementary material of this article.
