# Supplementary material for: Atomistic Insights into the Origin of High‐Performance Thermoelectric Response in Hybrid Perovskites
Source: Adv Sci (Weinh). 2023 May 11;10(20):2300666. doi: 10.1002/advs.202300666 (PMC10369277; doi:10.1002/advs.202300666)
Supplement: Supplementary file 1 — Supporting Information [file ADVS-10-2300666-s001.pdf]

ADVANCED SCIENCE
Open Access

Supporting Information

for *Adv. Sci.*, DOI 10.1002/advs.202300666

Atomistic Insights into the Origin of High-Performance Thermoelectric Response in Hybrid Perovskites

Wen Shi, Mingjia Yao, Xiaomei Wu, Tingxia Zhou, Xue Yong, Tianqi Deng, Huili Ma and Jinyang Xi**

Supporting Information

Atomistic Insights into the Origin of High-Performance Thermoelectric Response in Hybrid Perovskites

Wen Shi,[*] Mingjia Yao, Xiaomei Wu, Tingxia Zhou, Xue Yong, Tianqi Deng, Huili Ma, and Jinyang Xi[*]

Prof. W. Shi, X. M. Wu, T. X. Zhou
School of Chemistry, Sun Yat-sen University, Guangzhou, 510006, China
E-mail: shiw59@mail.sysu.edu.cn
M. J. Yao, Prof. J. Y. Xi
Materials Genome Institute, Shanghai University, Shanghai, 200444, China
E-mail: jinyangxi@t.shu.edu.cn
Dr. X. Yong
Department of Chemistry, The University of Sheffield, Brook Hill, Sheffield S3 7HF, United Kingdom
Dr. T. Q. Deng
State Key Laboratory of Silicon Materials, School of Materials Science and Engineering, Zhejiang University, Hangzhou, Zhejiang, 310027, China
Institute of Advanced Semiconductors & Zhejiang Provincial Key Laboratory of Power Semiconductor Materials and Devices, ZJU-Hangzhou Global Scientific and Technological Innovation Center, Hangzhou, Zhejiang, 311200, China
Prof. H. L. Ma
State Key Laboratory of Flexible Electronics & Institute of Advanced Materials, Nanjing Tech University, Nanjing, Jiangsu, 211816, China
Prof. J. Y. Xi
Zhejiang Laboratory, Hangzhou, Zhejiang, 311100, China

Table of Contents

Section S1. Computational Details

Section S2. Thermoelectric (TE) Power Factor and Figure of Merit

Section S3. Thermal Transport Properties

Section S4. Atomistic-Level Origin of Anharmonicity

Section S5. Charge Transport from the Standpoint of Electronic Structure and Lattice Dynamics

Section S6. Supporting References

Section S1. Computational Details

Model setup and geometric structural optimizations: We build the initial crystallographic structural models for α-$FAPbI_3$, δ-$FAPbI_3$ (FA = formamidinium), and $CsPbI_3$ based on the recently published works (Table S1). The atomic positions are optimized by the projector augmented-wave (PAW) method[1] with the Perdew-Burke-Ernzerhof (PBE) exchange-correlation functional[2] with the Grimme's D3 dispersion correction[3] in Vienna *Ab initio* Simulation Package (VASP, version 6.2.1)[4]. The convergence criterion of the total energy is 10^{-5} eV, and the force on each atom is smaller than 0.01 eV $Å^{-1}$. The cutoff radius for pair interactions are 50 Å. In this work, we concentrate on the room-temperature *p*-type TE transport properties for these materials.

Table S1. Lattice parameters of the initial crystallographic structures we used for α-$FAPbI_3$, δ-$FAPbI_3$, and $CsPbI_3$, respectively.

	a (Å)	b (Å)	c (Å)	α (°)	β (°)	γ (°)	V ($Å^3$)	ρ (g cm^{-3})	Ref.
α-$FAPbI_3$	9.00	9.00	12.7	90.0	90.0	90.0	1.03×10^3	4.08	[5]
δ-$FAPbI_3$	8.66	8.66	7.90	90.0	90.0	120	513	4.10	[5]
$CsPbI_3$	8.69	8.87	12.6	90.0	90.0	90.0	975	4.91	[6]

Einstein relationship for lattice thermal conductivity based on equilibrium ab initio MD simulations: To check the reliability of our simulated trajectories, we first extract and analyze a series of critical physical quantities, including temperature evolution with time (Figure S1), total energy and kinetic energy evolution with time (Figure S2 and Table S2), and force on each element evolution with time (Figure S3 and Table S3). On the basis of our statistical results, it is demonstrated that our *ab initio* MD trajectories for crystalline α-$FAPbI_3$, δ-$FAPbI_3$, and $CsPbI_3$ are reasonable.

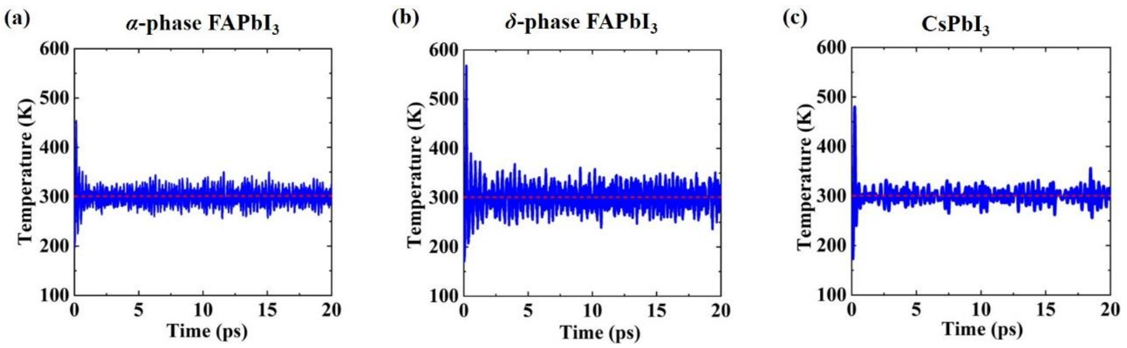

Figure S1. Temperature evolution with time for (**a**) α-$FAPbI_3$, (**b**) δ-$FAPbI_3$, and (**c**) $CsPbI_3$ single crystals, respectively based on the *ab initio* MD simulations.

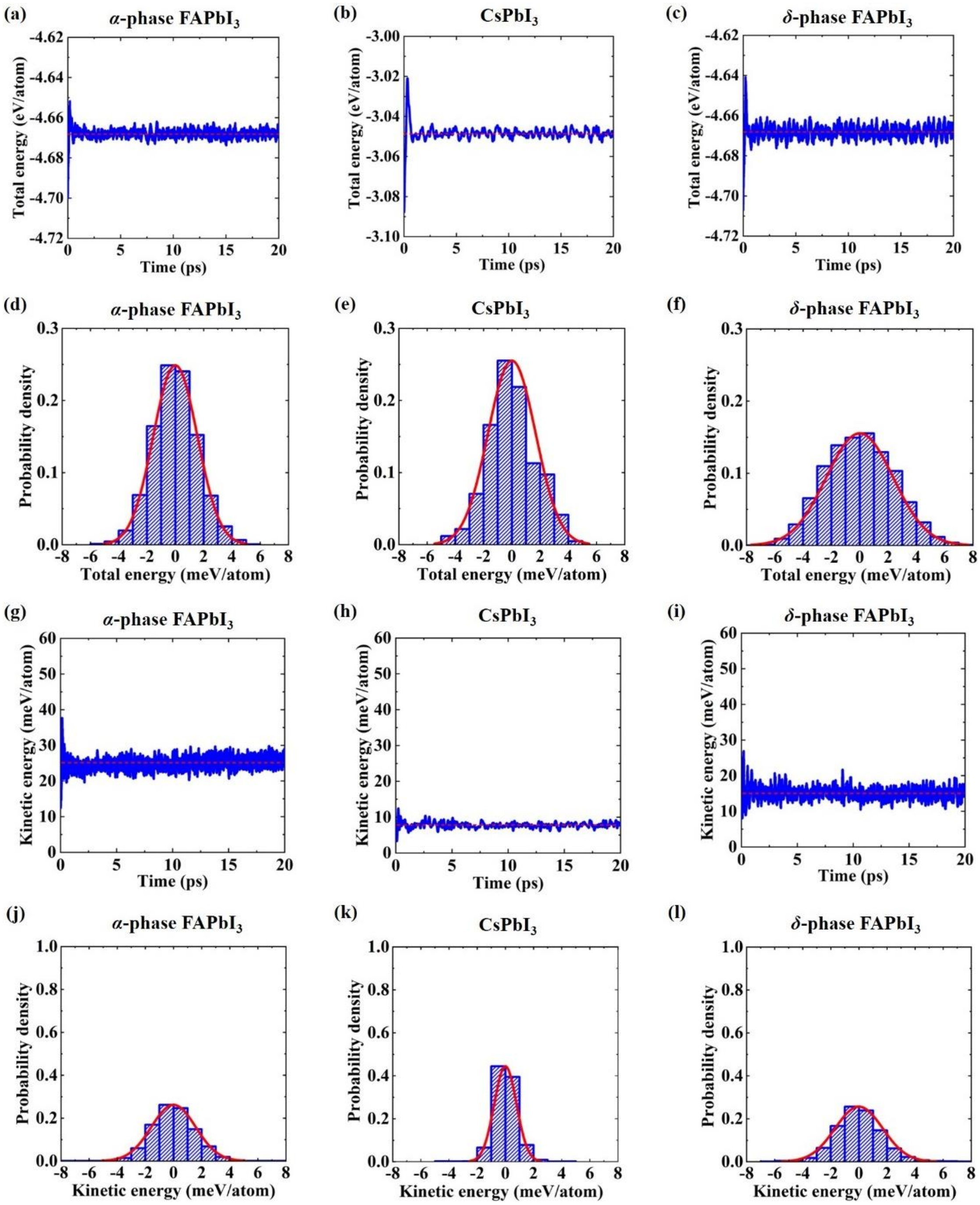

Figure S2. Total energy evolution with time for (**a**) α-$FAPbI_3$, (**b**) $CsPbI_3$, and (**c**) δ-$FAPbI_3$ single crystals, respectively based on the *ab initio* MD simulations at room temperature. Distribution of total energy for (**d**) α-$FAPbI_3$, (**e**) $CsPbI_3$, and (**f**) δ-$FAPbI_3$ single crystals, respectively at room temperature. Kinetic energy evolution with time for (**g**) α-$FAPbI_3$, (**h**) $CsPbI_3$, and (**i**) δ-$FAPbI_3$ single crystals, respectively based on the *ab initio* MD simulations at room temperature. Distribution of kinetic energy for (**j**) α-$FAPbI_3$, (**k**) $CsPbI_3$, and (**l**) δ-$FAPbI_3$ single crystals, respectively at room temperature. The red lines in (**d**−**f**) and (**j**−**l**) are the fitted Gaussian distributions.

Table S2. Standard deviation of the total energy (ΔE_{total}), kinetic energy ($\Delta E_{\text{kinetic}}$), and potential energy ($\Delta E_{\text{potential}}$) of the transient configurations for crystalline α-$FAPbI_3$, δ-$FAPbI_3$, and $CsPbI_3$, respectively at room temperature based on the *ab initio* MD simulations. Here, $\Delta E = \sqrt{\frac{1}{N}\sum_{i=1}^{N}(E_i - \bar{E})^2}$, where N is the number of transient configurations; E_i is the energy of each transient configuration, and $\bar{E}$ is the average energy of these transient configurations.

	ΔE_{total} (meV/atom)	$\Delta E_{\text{kinetic}}$ (meV/atom)	$\Delta E_{\text{potential}}$ (meV/atom)
α-$FAPbI_3$	2.13	0.606	2.08
δ-$FAPbI_3$	3.24	0.325	3.13
$CsPbI_3$	3.76	0.284	2.43

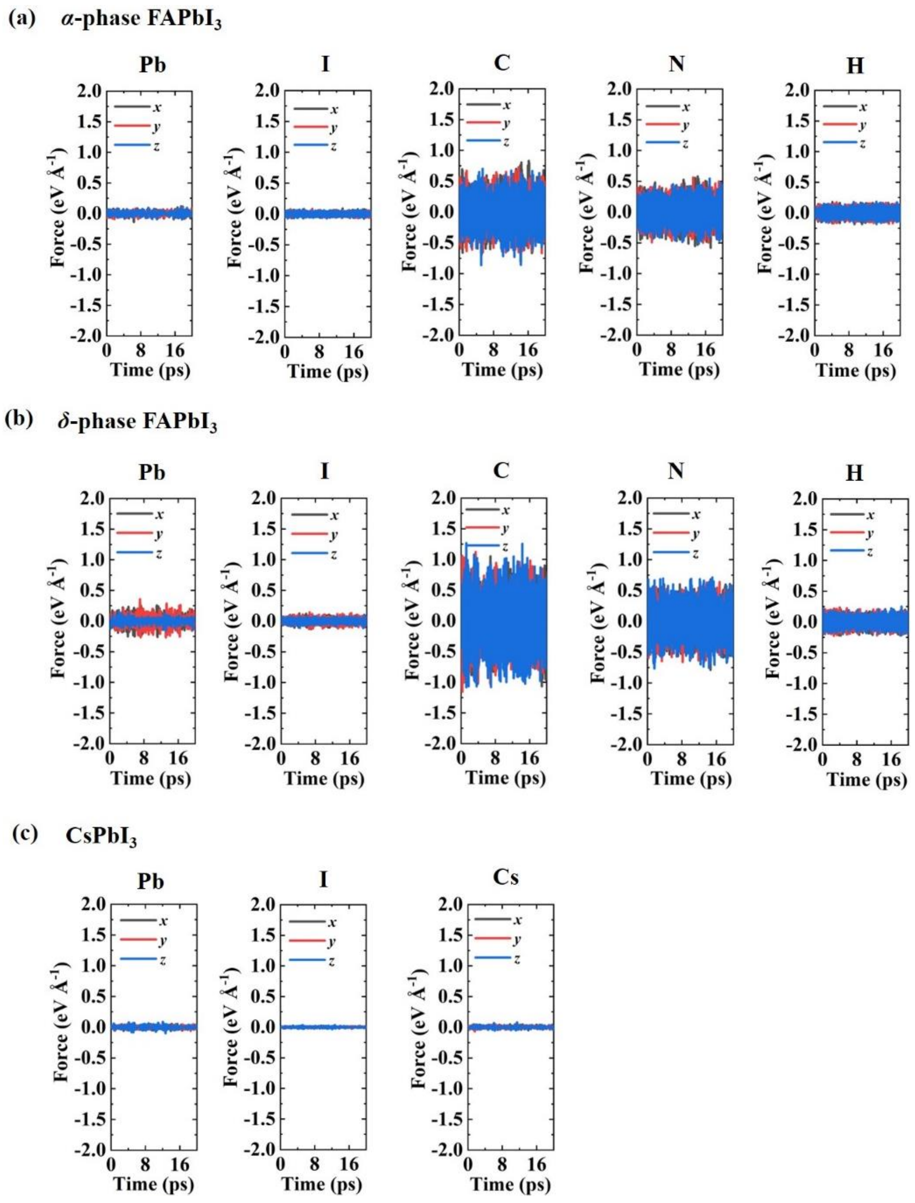

Figure S3. Average force of each element evolution with time for (**a**) α-$FAPbI_3$, (**b**) δ-$FAPbI_3$, and (**c**) $CsPbI_3$ single crystals, respectively at room temperature based on the *ab initio* MD simulations.

Table S3. Standard deviation of force (ΔF) for each element for crystalline α-$FAPbI_3$, δ-$FAPbI_3$, and $CsPbI_3$, respectively at room temperature based on the *ab initio* MD simulations. Here, $\Delta F_\alpha = \sqrt{\frac{1}{N}\sum_{i=1}^{N}(F_{\alpha i} - \overline{F_\alpha})^2}$, where N is the number of transient configurations; $F_{\alpha i}$ is the force on the i-th atom; $\overline{F_\alpha}$ is the average force of the specified atom for these transient configurations, and α is the cartesian direction. For the same element, the average standard deviations of force are present.

ΔF (eV Å^{-1})	Pb			I			C			N			H		
	x	*y*	*z*	*x*	*y*	*z*	*x*	*y*	*z*	*x*	*y*	*z*	*x*	*y*	*z*
α-$FAPbI_3$	0.036	0.033	0.034	0.022	0.021	0.022	0.21	0.19	0.20	0.14	0.14	0.14	0.050	0.050	0.049
δ-$FAPbI_3$	0.087	0.091	0.046	0.035	0.040	0.026	0.27	0.29	0.37	0.18	0.18	0.22	0.062	0.063	0.060

ΔF (eV Å^{-1})	Pb			I			Cs		
	x	*y*	*z*	*x*	*y*	*z*	*x*	*y*	*z*
$CsPbI_3$	0.022	0.020	0.028	0.0092	0.0084	0.011	0.018	0.018	0.019

Unlike the Green-Kubo formula computing the lattice thermal conductivity from the autocorrelation function of the heat current, in this scheme, the lattice thermal conductivity can be expressed as the diffusion of the energy momentum ($\boldsymbol{R}$).[7] Its expression is displayed in the “Methods Section” of the main text. It has been demonstrated that this definition of energy momentum is valid for *N*-body potentials.[7] Moreover, it should be noted that this method is equivalent to Green-Kubo relation and non-perturbative. Owing to the anharmonic energy surface obtained from the MD trajectory, all the anharmonic interactions are naturally included Therefore, this method is quite suitable for intrinsically highly anharmonic systems, such as our studied materials.

The calculated energy momentum for crystalline α-$FAPbI_3$, δ-$FAPbI_3$, and $CsPbI_3$ are displayed in Figure S4. In addition, the heat current ($\boldsymbol{J}$) can be calculated from the time derivative of the energy momentum[7], that is,

$$\boldsymbol{J} = \frac{\mathrm{d}\boldsymbol{R}}{\mathrm{d}t}, \tag{S1}$$

and the related results are shown in Figure S5 and Table S4. Thus, we can obtain the heat current autocorrelation function $[\mathrm{Cor}(t)]$ via the formula[7]

$$\mathrm{Cor}(t)_{\alpha\beta} = \frac{\langle J_\alpha(t) J_\beta(0)\rangle}{\langle J_\alpha(0) J_\beta(0)\rangle}, \tag{S2}$$

and the related results are displayed in Figure S6. The exponential decay form of heat current autocorrelation function can be expressed as a sum of $3n$ damped harmonic oscillators. The phonon lifetime ($\langle \tau_{\text{phonon}} \rangle$) is extracted from the linewidth of the Fourier transform of the heat current autocorrelation function at low-frequency region. Accordingly, the phonon mean free path ($\langle l_{\text{phonon}} \rangle$) is estimated by the formula

$$\langle l_{\text{phonon}} \rangle = v_{\text{L}} \langle \tau_{\text{phonon}} \rangle, \tag{S3}$$

Here, v_{L} is the group velocity of the longitudinal acoustic mode computed through the formula

$$v_{\text{L}} = \sqrt{C/\rho}, \tag{S4}$$

where C and ρ are the elastic constant and mass density, respectively. The details for computing the elastic constant are exhibited below.

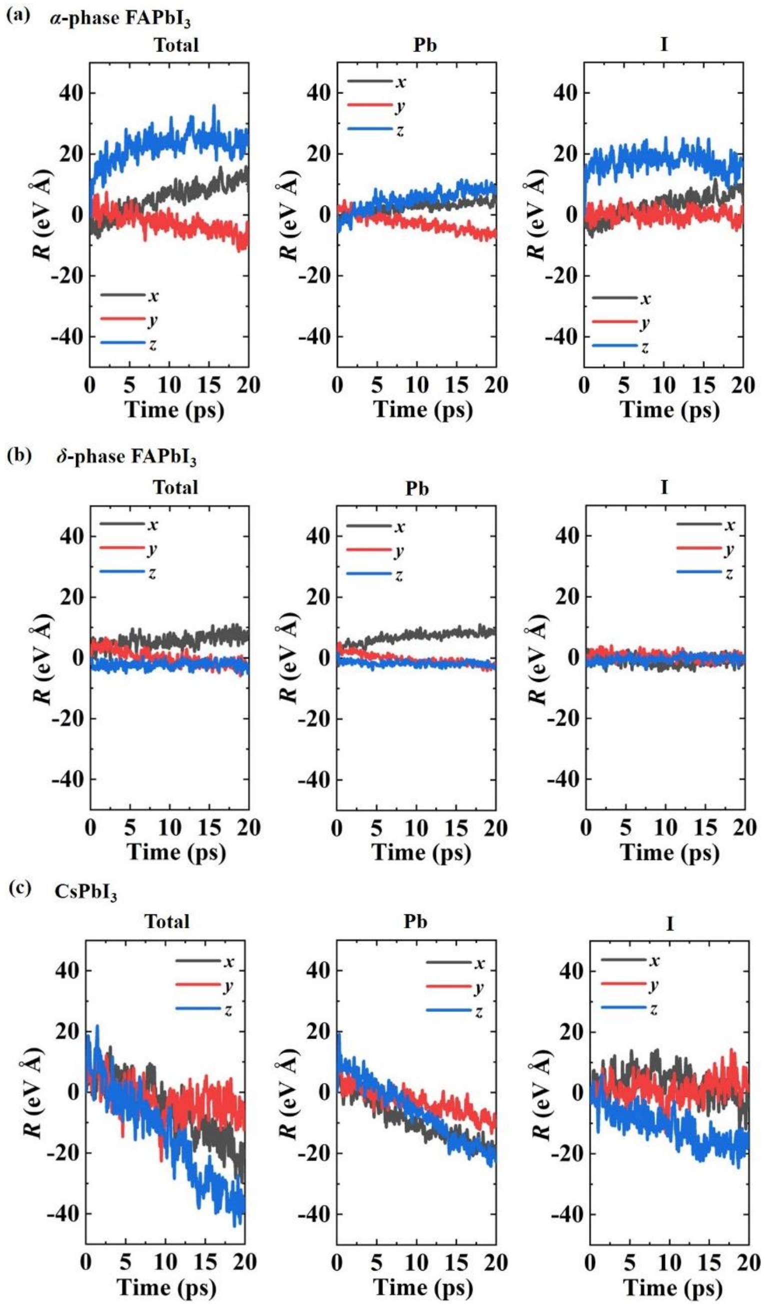

Figure S4. Relationship between the energy momentum (***R***) and the time along the *x* (black), *y* (red), and *z* (blue) directions for (**a**) α-$FAPbI_3$, (**b**) δ-$FAPbI_3$, and (**c**) $CsPbI_3$ single crystals, respectively at room temperature based on the *ab initio* MD simulations. The contributions of lead and iodine elements to the energy momentum are also exhibited.

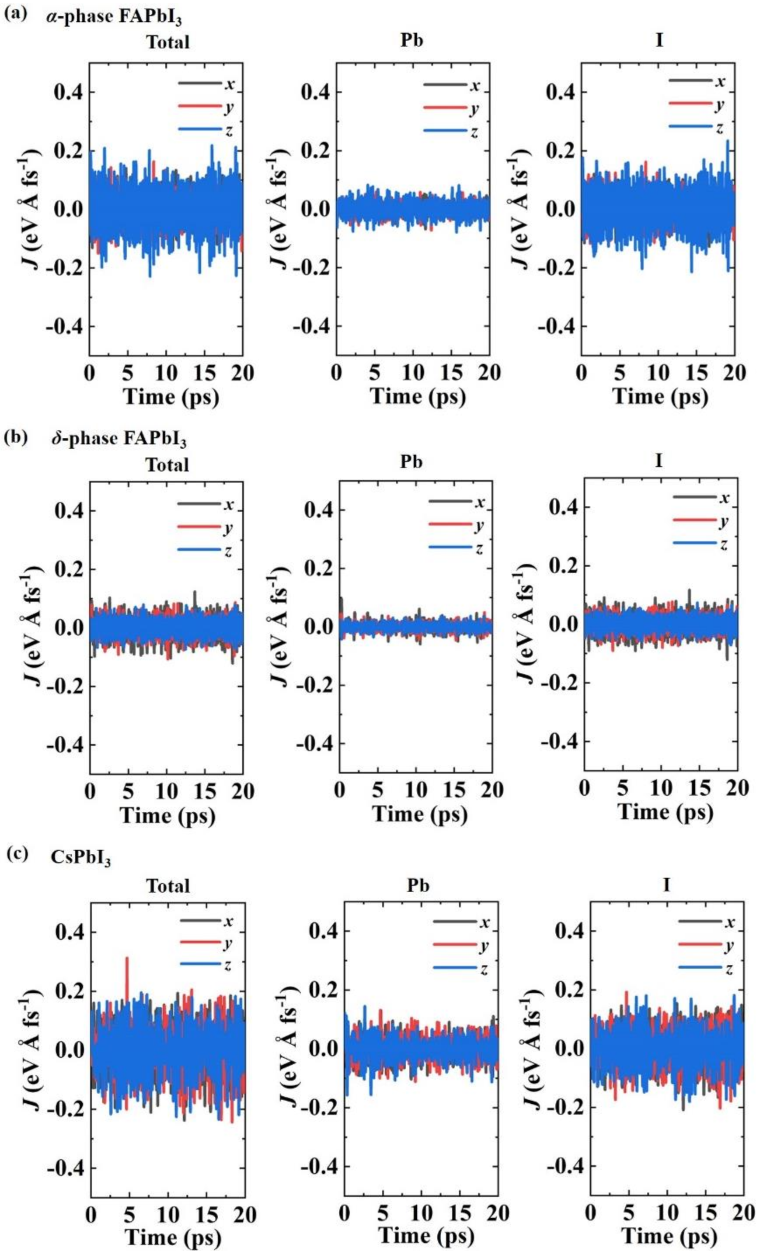

Figure S5. Relationship between the heat current ($\boldsymbol{J}$) and the time along the *x* (black), *y* (red), and *z* (blue) directions for (**a**) α-$FAPbI_3$, (**b**) δ-$FAPbI_3$, and (**c**) $CsPbI_3$ single crystals, respectively at room temperature based on the *ab initio* MD simulations. The contributions of lead and iodine elements to the heat current are also displayed.

Table S4. Standard deviation of heat current (ΔJ) for organic and inorganic components for crystalline α-$FAPbI_3$, δ-$FAPbI_3$, and $CsPbI_3$, respectively at room temperature based on the *ab initio* MD simulations. Here, $\Delta J_\alpha = \sqrt{\frac{1}{N}\sum_{i=1}^{N}(J_{\alpha i} - \overline{J_\alpha})^2}$, where N is the total simulation time; $J_{\alpha i}$ is the heat current at the i-th timestep; $\overline{J_\alpha}$ is the average heat current, and α is the cartesian direction.

ΔJ (eV Å fs^{-1})	Pb			I			Total		
	x	y	z	x	y	z	x	y	z
α-$FAPbI_3$	0.018	0.019	0.026	0.035	0.037	0.054	0.038	0.041	0.060
δ-$FAPbI_3$	0.016	0.013	0.012	0.027	0.022	0.019	0.031	0.026	0.023
$CsPbI_3$	0.036	0.037	0.038	0.060	0.058	0.061	0.075	0.075	0.077

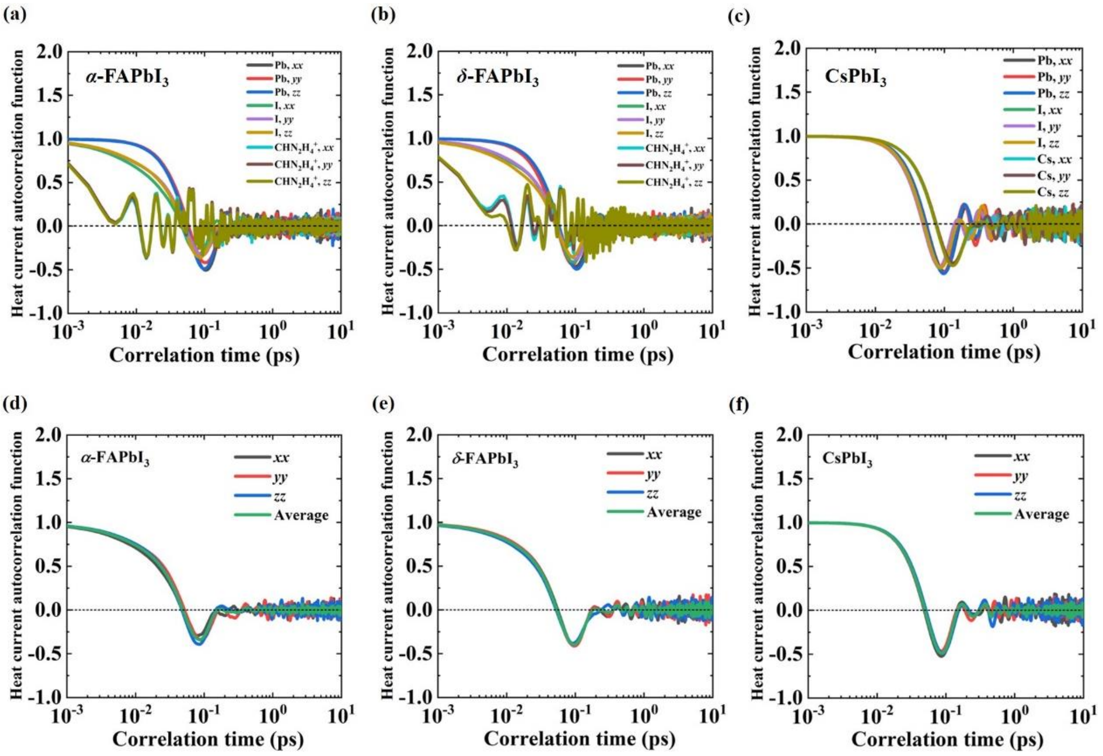

Figure S6. Heat current autocorrelation function for organic [$(CHN_2H_4)^+$] and inorganic (Pb and I) components along the x, y, and z directions for (**a**) α-$FAPbI_3$, (**b**) δ-$FAPbI_3$, and (**c**) $CsPbI_3$ single crystals, respectively at room temperature based on the *ab initio* MD simulations. Direction-averaged heat current autocorrelation function (green) and contributions from the x (black), y (red), and z (blue) components for (**d**) α-$FAPbI_3$, (**e**) δ-$FAPbI_3$, and (**f**) $CsPbI_3$ single crystals, respectively at room temperature based on the *ab initio* MD simulations.

Moreover, by analyzing the atomic velocities in MD trajectories (Figure S7 and Table S5), the vibrational density of states (DOS) [$f(\omega)$] can be computed by the Fourier transform of velocity autocorrelation function [$\mathrm{Cor}(t)$] (Figure S8), that is,

$$f(\omega) = \mathrm{Re}\left[\int_0^{\infty} \mathrm{Cor}(t) \mathrm{e}^{i\omega t} \mathrm{d}t\right], \tag{S5}$$

where $\mathrm{Cor}(t) = \langle \sum_i^n \boldsymbol{v}_i(t) \cdot \boldsymbol{v}_i(0) \rangle / \langle \sum_i^n \boldsymbol{v}_i(0)^2 \rangle$; $\boldsymbol{v}_i(t)$ is the velocity of the i-th atom at time t; ω is the frequency, and the angular brackets stand for the ensemble average. According to the definition of the constant volume heat capacity per volume, that is, $c_\mathrm{V} = \frac{1}{V}\left(\frac{\partial U}{\partial T}\right)_\mathrm{V}$, and

considering the thermal population of the vibrational modes, the heat capacity per volume at constant volume can be calculated by the equation[8]

$$c_{\mathrm{V}} = \frac{k_{\mathrm{B}}}{V}\int f(\omega)\frac{\left(\frac{\hbar\omega}{k_{\mathrm{B}}T}\right)^{2}\exp\left(\frac{\hbar\omega}{k_{\mathrm{B}}T}\right)}{\left[\exp\left(\frac{\hbar\omega}{k_{\mathrm{B}}T}\right)-1\right]^{2}}\mathrm{d}\omega, \qquad (S6)$$

where k_{B} is Boltzmann constant; V is volume, and $\hbar$ is reduced Planck constant.

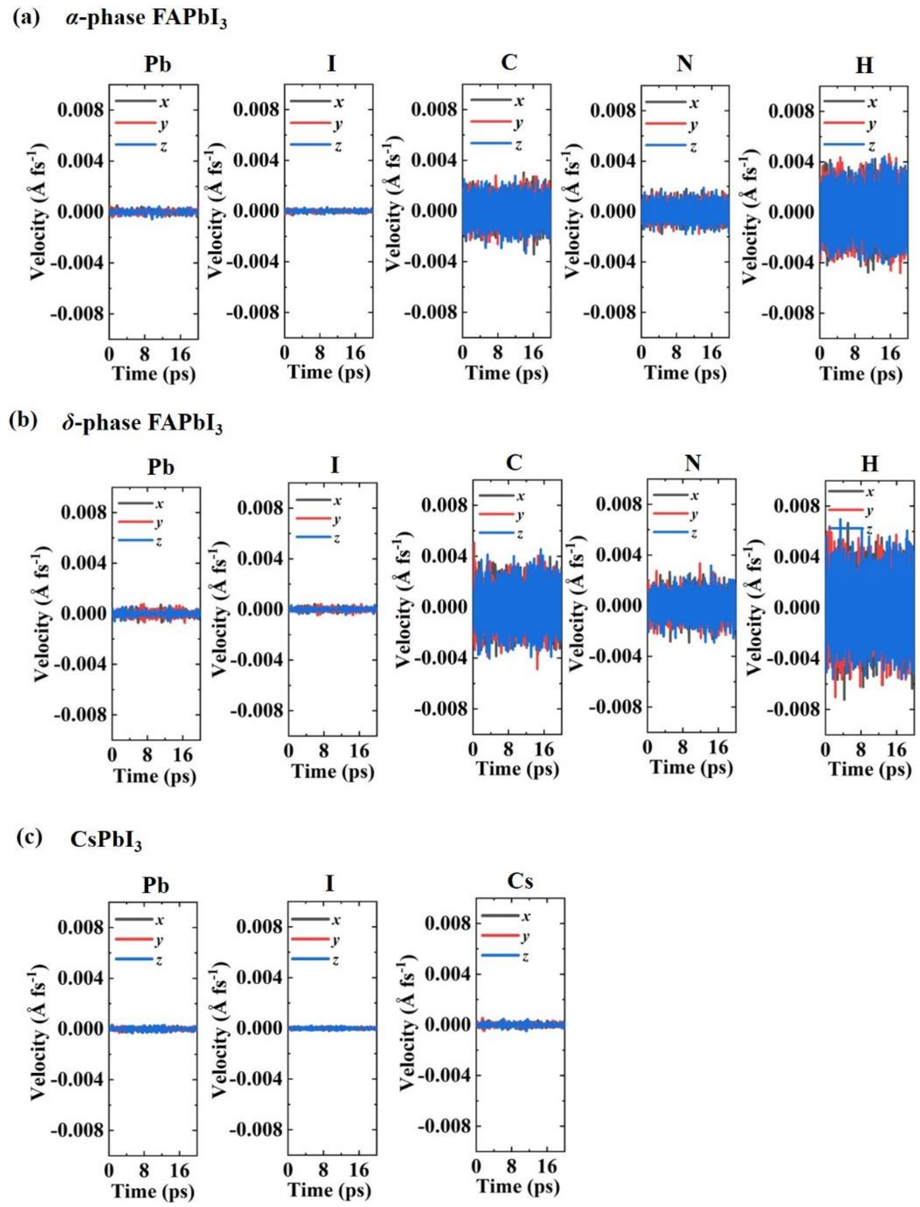

Figure S7. Average atomic velocity of each element evolution with time for (**a**) α-$FAPbI_3$, (**b**) δ-$FAPbI_3$, and (**c**) $CsPbI_3$ single crystals, respectively at room temperature based on the *ab initio* MD simulations.

Table S5. Standard deviation of velocity (Δv) for each element for crystalline α-$FAPbI_3$, δ-$FAPbI_3$, and $CsPbI_3$, respectively at room temperature based on the *ab initio* MD simulations. Here, $\Delta v_\alpha = \sqrt{\frac{1}{N}\sum_{i=1}^{N}(v_{\alpha i} - \overline{v_\alpha})^2}$, where N is the number of transient configurations; $v_{\alpha i}$ is the velocity of the i-th atom; $\overline{v_\alpha}$ is the average velocity of the specified atom for these transient configurations, and α is the cartesian direction. For the same element, the average standard deviations of velocity are present.

Δv (10^{-4} Å fs^{-1})	Pb			I			C			N			H		
	x	y	z	x	y	z	x	y	z	x	y	z	x	y	z
α-$FAPbI_3$	1.64	1.40	1.67	0.986	0.802	0.909	8.49	7.65	7.96	5.30	5.02	5.29	1.25	1.28	1.31
δ-$FAPbI_3$	2.31	2.41	2.14	1.27	1.43	1.23	1.12	1.11	1.23	7.52	7.39	7.79	1.69	1.67	1.67

Δv (10^{-4} Å fs^{-1})	Pb			I			Cs		
	x	y	z	x	y	z	x	y	z
$CsPbI_3$	0.930	0.920	1.13	0.581	0.548	0.652	1.36	1.36	1.39

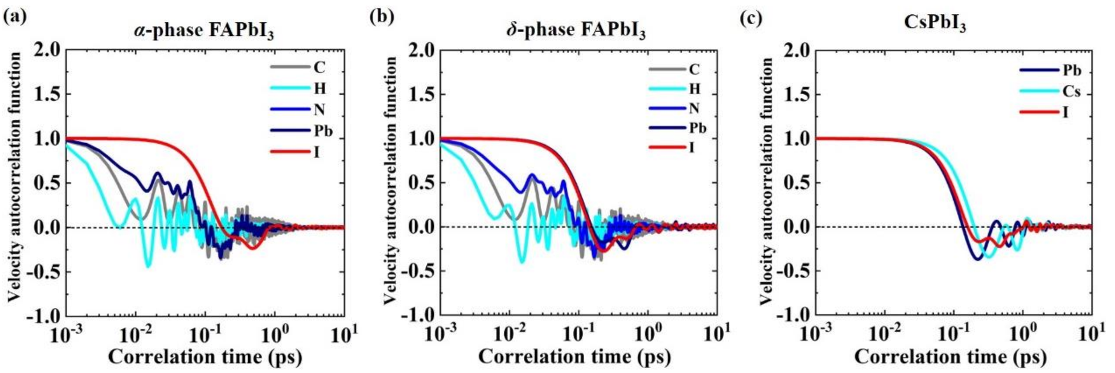

Figure S8. Velocity autocorrelation function for each element for (**a**) α-$FAPbI_3$, (**b**) δ-$FAPbI_3$, and (**c**) $CsPbI_3$ single crystals, respectively at room temperature based on the *ab initio* MD simulations.

Phonon Boltzmann transport equation for lattice thermal conductivity based on equilibrium ab initio MD simulations: The phonon Boltzmann transport equation[9] has the form

$$\kappa_{\mathrm{L}\,\alpha\beta} = \frac{1}{N_{\boldsymbol{q}}\Omega}\sum_{\boldsymbol{q},s} c_{\boldsymbol{q},s} v^{\alpha}_{\boldsymbol{q},s} v^{\beta}_{\boldsymbol{q},s} \tau_{\boldsymbol{q},s}. \tag{S7}$$

Here, $N_{\boldsymbol{q}}$ is the number of q points; Ω is the unit cell volume; $c_{\boldsymbol{q},s} = \frac{k_{\mathrm{B}}\left(\frac{\hbar\omega_{\boldsymbol{q},s}}{k_{\mathrm{B}}T}\right)^2 \exp\left(\frac{\hbar\omega_{\boldsymbol{q},s}}{k_{\mathrm{B}}T}\right)}{\left[\exp\left(\frac{\hbar\omega_{\boldsymbol{q},s}}{k_{\mathrm{B}}T}\right)-1\right]^2}$ is the mode heat capacity; $\boldsymbol{v}_{\boldsymbol{q},s} = \frac{\mathrm{d}\omega_{\boldsymbol{q},s}}{\mathrm{d}\boldsymbol{q}}$ is the phonon group velocity, and $\tau_{\boldsymbol{q},s}$ is the phonon lifetime. It is known that the phonon frequencies can be attained by diagonalizing the dynamical matrix, and the latter is related to the harmonic force constants defined as the second derivatives of the potential energy with respect to small atomic displacements from equilibrium.

Furthermore, assuming the dominant role of three-phonon processes in the phonon lifetime, the scattering matrix element is related to the third-order force constants. To extract the second- and third-order force constants for $CsPbI_3$ single crystal, we utilize the temperature-dependent effective potential method[9] to map the anharmonic potential obtained from Born-Oppenheimer MD simulations onto a model Hamiltonian. Thus, both the room-temperature phonon band, group velocity, phonon lifetime, and lattice thermal conductivity are simultaneously achieved (Figure S7).

Here, after reaching equilibrium in MD simulations, we extract the position and force of each atom in each step to evaluate the harmonic and third-order force constants. The cutoff radii for the second- and third-order force constants are 15 and 5 Å, respectively. A *q*-mesh of 10×10×10 is used to calculate the lattice thermal conductivity. It is worth noting that in this method, the temperature-caused anharmonic effect on the lattice dynamics can be naturally included.

In this work, we employ Einstein relation to calculate the room-temperature lattice thermal conductivity of all the systems in conjunction with equilibrium *ab initio* MD. Unlike phonon Boltzmann transport equation, this method is equivalent to Green-Kubo relation and non-perturbative. Moreover, all the anharmonic interactions are naturally included due to the anharmonic energy surface obtained from MD trajectory. To confirm the rationality of the thermal transport properties calculated by the Einstein relationship, we take the crystalline $CsPbI_3$ as an example to compute its room-temperature lattice thermal conductivity by utilizing phonon Boltzmann transport equation. Importantly, our results demonstrate that the lattice thermal conductivities of crystalline $CsPbI_3$ computed by Einstein relationship agree with those obtained by phonon Boltzmann transport equation, evidently proving the rationality of our *ab initio* MD methodology for heat transport, which also satisfactorily achieves our purpose of performing the validation studies. In principle, phonon Boltzmann transport equation can be used to evaluate the lattice thermal conductivity of α- and δ-$FAPbI_3$ single crystals, and there is no restriction.

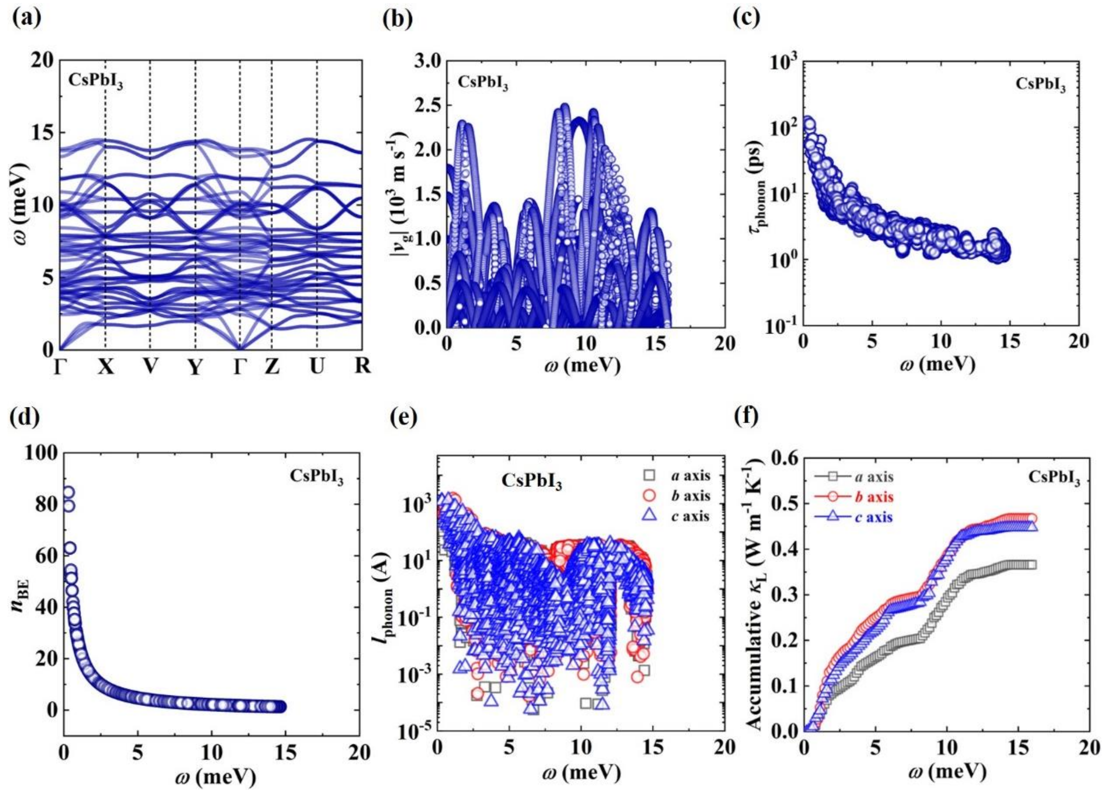

Figure S9. Room-temperature (**a**) phonon distribution, (**b**) phonon group velocity ($|v_{\mathrm{g}}|$), (**c**) frequency-dependent phonon lifetime (τ_{phonon}), (**d**) frequency-dependent phonon occupation number (n_{BE}), (**e**) *a*- (black square), *b*- (red circle), and *c*-axis (blue triangle) frequency-dependent phonon mean free path (l_{phonon}), and (**f**) *a*- (black square), *b*- (red circle), and *c*-axis (blue triangle) accumulative lattice thermal conductivity (κ_{L}) for $CsPbI_3$ single crystal based on *ab initio* MD simulations and phonon Boltzmann transport equation. The reciprocal coordinates of high symmetry ***k***-points in the first Brillouin zone are **Γ** = (0, 0, 0), **X** = (0.5, 0, 0), **V** = (0.5, 0.5, 0), **Y** = (0, 0.5, 0), **Z** = (0, 0, 0.5), **U** = (0.5, 0, 0.5), and **R** = (0.5, 0.5, 0.5), respectively. Here, $n_{\mathrm{BE}} = \frac{1}{\exp[\hbar\omega/(k_{\mathrm{B}}T)]-1}$ is the Bose-Einstein distribution function.

Orbital projected band structure: By projecting the Kohn-Sham wave functions onto the spherical harmonics centered at the position of the ions based on the formula, $P_{Nlmn\boldsymbol{k}} = \langle Y_{lm}^{N} | \phi_{n\boldsymbol{k}} \rangle$, we calculate the orbital projected band structures. Here, Y_{lm}^{N} and $\phi_{n\boldsymbol{k}}$ are the spherical harmonics centered at ion index N with angular moment l and magnetic quantum number m, and the Kohn-Sham wave functions with band index n and wave vector $\boldsymbol{k}$, respectively.[10]

Fröhlich polaron model for electron-optical phonon interactions: This model assumes that the spatial extension of polaron is large compared to the lattice constant of the solid, and the longitudinal optical phonons of interest for the interaction are the long-wavelength phonons

with constant frequency. Recently, on the basis of multiphonon Raman scattering and THz time-domain spectroscopy, it has been proven that the Fröhlich interaction caused by a longitudinal optical phonon mode governs the electron-lattice coupling in lead halide perovskites.[11] By using temperature-dependent photoluminescence spectra, the researchers have demonstrated that scattering from longitudinal optical phonons *via* the Fröhlich interaction is the dominant source of electron-phonon coupling near room temperature for formamidinium and methylammonium perovskites.[12] Hence, Fröhlich polaron model is suitable for our studied systems.

In this model, the strength of electron-optical phonon coupling is measured by a dimensionless Fröhlich parameter,

$$\alpha_{\mathrm{e-ph}} = \frac{\mathrm{e}^2}{\hbar}\frac{1}{4\pi\varepsilon}\left(\frac{1}{\varepsilon_\infty}-\frac{1}{\varepsilon_0}\right)\sqrt{\frac{m^*}{2\hbar\omega_{\mathrm{eff}}}}, \quad (S8)$$

where e is the elementary charge; ε is the dielectric constant of vacuum (8.85×10^{-12} C V^{-1} m^{-1}); ε_∞ is the high-frequency dielectric constant; ε_0 is the static dielectric constant, m^* is the bare hole effective mass, and ω_{eff} is the effective frequency of longitudinal optical phonon.[13,14] Here, the effective frequency of longitudinal optical phonon is extracted from the vibrational density of states based on the aforementioned *ab initio* MD simulations, and the related results are summarized in Table S6. To obtain the relative high frequency and static dielectric constants, the frequency-dependent dielectric properties are studied based on first-principles calculations by using the PAW method[1] with the PBE functional[2] in VASP[4], and the related results are displayed in Figure S10 and Table S7. The ionic contributions are evaluated based on density functional perturbation theory (DFPT)[15], and the electronic ones are achieved by the independent particle approximation[15]. The convergence criterion of the total energy is set to be 10^{-8} eV. Due to the less dispersive characteristic of low-frequency optical modes, it can be regarded that the relaxation time caused by electron-optical phonon interactions only depends on the vibrational frequency. The extracted parameters used in Fröhlich polaron model are shown in Table S8.

Table S6. Vibrational frequencies of optical modes (ω_i) extracted from the vibrational DOS for crystalline α-$\mathrm{FAPbI_3}$, δ-$\mathrm{FAPbI_3}$, and $\mathrm{CsPbI_3}$, respectively at room temperature. The effective frequency of optical phonon (ω_{eff}) is defined as $\omega_{\mathrm{eff}} = \frac{1}{n}\sum_i^n \omega_i$.

	ω_1 (meV)	ω_2 (meV)	ω_3 (meV)	ω_4 (meV)	ω_{eff} (meV)
α-$\mathrm{FAPbI_3}$	3.31	6.20	8.55	10.8	7.20
δ-$\mathrm{FAPbI_3}$	2.34	4.69	6.48	10.9	6.10

$CsPbI_3$	3.45	7.44	10.3	14.2	8.86

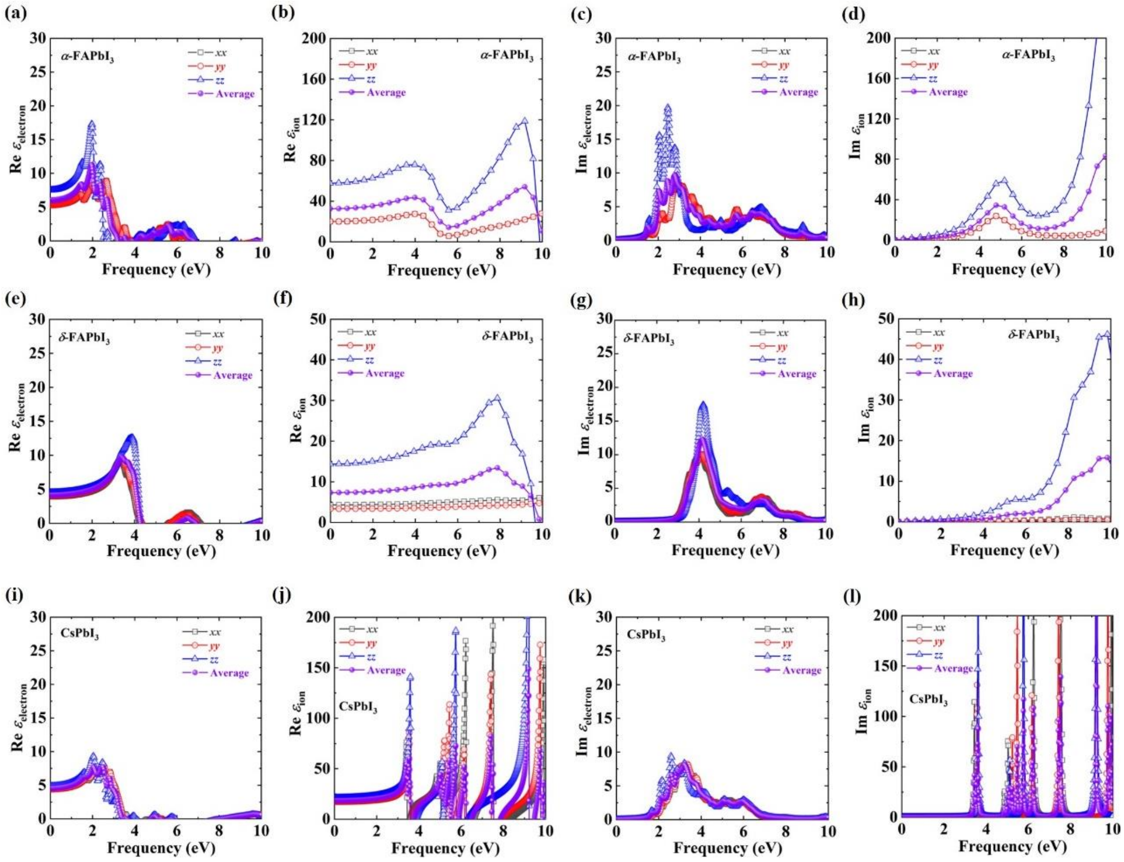

Figure S10. Frequency-dependent relative dielectric constant along the *x* (black square), *y* (red circle), and *z* (blue triangle) directions for (**a**–**d**) α-$FAPbI_3$, (**e**–**h**) δ-$FAPbI_3$, and (**i**–**l**) $CsPbI_3$ single crystals, respectively. The direction-averaged relative dielectric constants (purple circle) are also shown in the figure. Here, $\varepsilon_{\mathrm{electron}}$ and $\varepsilon_{\mathrm{ion}}$ are the contributions of electrons and ions to the dielectric constant, respectively; Re and Im stand for the real and imaginary parts, respectively.

Table S7. Static (ε_0) and high-frequency dielectric constants (ε_∞) for α-$FAPbI_3$, δ-$FAPbI_3$, and $CsPbI_3$ single crystals, respectively.

	ε_0	ε_∞
α-$FAPbI_3$	38.6	6.04
δ-$FAPbI_3$	11.5	4.16
$CsPbI_3$	23.0	4.54

Table S8. Electron-optical phonon coupling ($\alpha_{\mathrm{e-ph}}$), variational parameters (v and w) at the lowest free energy of polaron (F), and polaron free energy for α-$FAPbI_3$, δ-$FAPbI_3$, and $CsPbI_3$ single crystals, respectively at room temperature.

	$\alpha_{\mathrm{e-ph}}$	v	w	F (meV)

α-$FAPbI_3$	2.81	25.6	21.7	−47.3
δ-$FAPbI_3$	7.68	35.9	22.4	−164
$CsPbI_3$	3.53	21.7	17.1	−67.3

Deformation potential (DP) model for electron-acoustic phonon interactions: This theory is modeled as the lattice dilation to approximate the acoustic phonon scattering. The elastic constant and the DP constant measured as the first-order derivative of band edge position with respective to the lattice dilation, are two key parameters in this theory. It is worth noting that DP theory has been widely used to investigate the electron-acoustic phonon interactions in crystalline organic molecular semiconductors[16], crystalline conducting polymers[17], and perovskite single crystals[18,19]. More importantly, these theoretical works validate the rationality of this model for studying electron-acoustic phonon interactions. Therefore, for our studied systems, it is suitable to employ DP theory to model their electron-acoustic phonon interactions.

To compute the elastic constant, we stretch the unit cell along the *a*, *b*, and *c* directions separately by ±0.5% and ± 1.0%, and then fit the total energy (E) of the deformed lattice with respect to the dilation ($\frac{\Delta l}{l_0}$) through the formula

$$\frac{E - E_0}{\Omega} = \frac{C_{ii}}{2}\left(\frac{\Delta l}{l_0}\right)^2, \tag{S9}$$

where Ω is the unit cell volume; E_0 and l_0 are the total energy and lattice parameter at equilibrium, respectively; Δl is the change of lattice parameters. To calculate the DP constant, we evaluate the band energies with the lattice deformation, and then fit the band edge to the dilation ($\frac{\Delta l}{l_0}$) through the formula,

$$E_1 = \frac{\Delta E_{\mathrm{VBM/CBM}}}{\Delta l / l_0}, \tag{S10}$$

where, $\Delta E_{\mathrm{VBM/CBM}}$ is the position change of valence band maximum or conduction band minimal (VBM/CBM) with the lattice deformation.[20,21] Herein, due to its insensitivity to the slight lattice deformation, the localized lowest energy level is treated as the energy reference to attain the absolute band energy changes of band edge.[22] The calculated DP constants and elastic constants for α-$FAPbI_3$, δ-$FAPbI_3$, and $CsPbI_3$ single crystals are summarized in Table S9.

Table S9. Hole DP constant (E_1) and elastic constant (C_{ii}) along the *a*, *b*, and *c* axes for α-$FAPbI_3$, δ-$FAPbI_3$, and $CsPbI_3$ single crystals, respectively.

	E_1 (eV)			C_{ii} (10^9 J m^{-3})		
	a	*b*	*c*	*a*	*b*	*c*
α-$FAPbI_3$	2.02	2.04	3.57	36.6	36.9	79.1
δ-$FAPbI_3$	0.170	0.604	0.802	19.6	33.5	56.8
$CsPbI_3$	2.86	0.062	1.59	21.7	36.4	55.0

Brooks-Herring approach for electron-charged impurity interactions: In this approach, the screened Coulomb potential between the charged impurities and charge carriers can be expressed as $V(r) = \frac{q_\mathrm{I}\mathrm{e}}{4\pi\varepsilon_\mathrm{r}\varepsilon_0 r}\exp\left(-\frac{r}{L_\mathrm{D}}\right)$, where q_I is the impurity charge; $L_\mathrm{D} = \sqrt{\frac{\varepsilon_\mathrm{r}\varepsilon_0 k_\mathrm{B}T}{e^2N}}$ is the screening length; N is the carrier concentration, and ε_r is the relative permittivity. Here, $\varepsilon_\mathrm{r} = \frac{1}{2}(\varepsilon_\infty + \varepsilon_0)$, and as mentioned above, both the ε_∞ and ε_0 are obtained from first-principles calculations. Additionally, we assume that the n_I impurities independently scatter the charge carriers in each unit cell.[10,23]

This model assumes that the scattering potential caused by the impurity center is treated as the screened Coulomb potential, and the Debye screening length is thus introduced. Recent published theoretical works have demonstrated that it is reliable to apply Brooks-Herring approach to probe the electron-charged impurity interactions in organic-based materials[23] and perovskite materials[24]. Consequently, this model is suitable for our studied systems.

Section 2. TE Power Factor and Figure of Merit

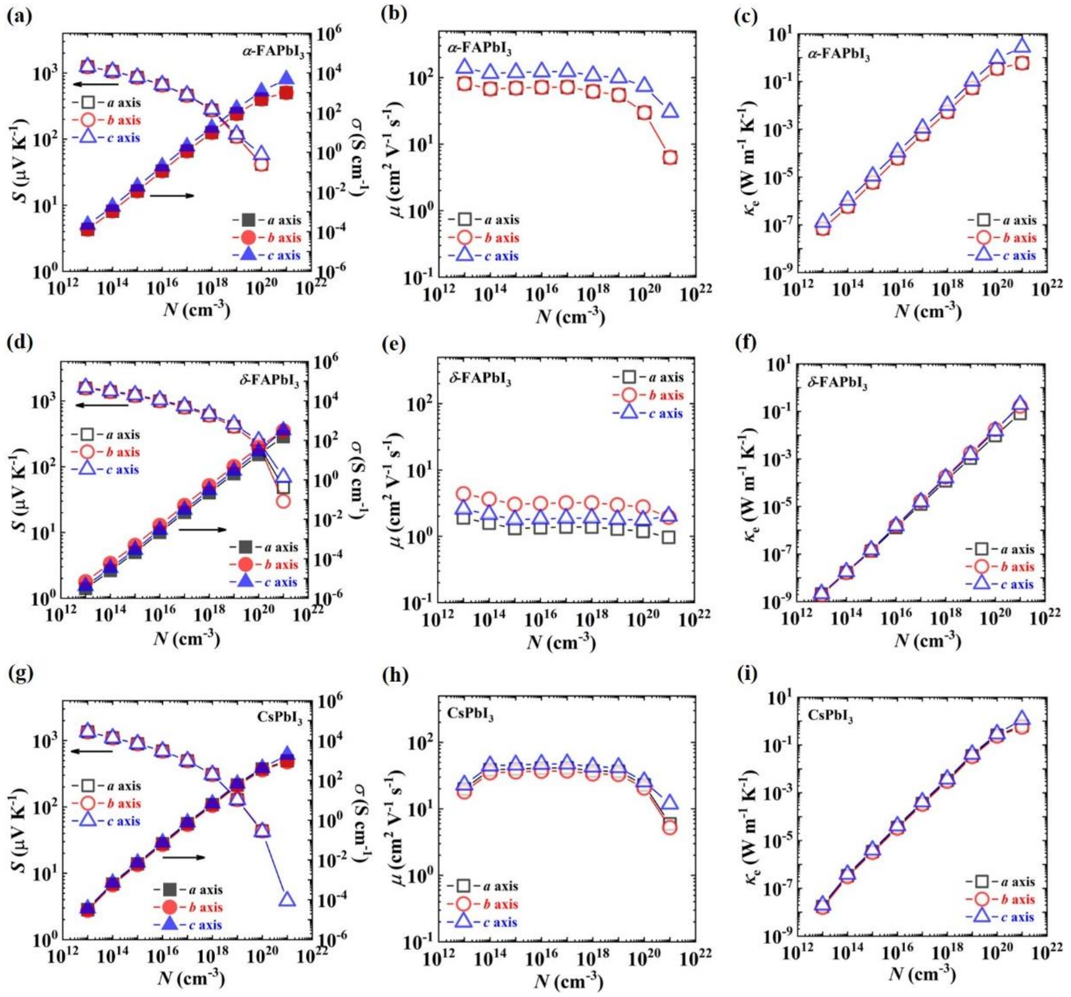

Figure S11. Room-temperature TE transport coefficients including Seebeck coefficient (S), conductivity (σ), mobility (μ), and electronic thermal conductivity (κ_e) along the a (black square), b (red circle) and c (blue triangle) axes, respectively for (**a**–**c**) α-$FAPbI_3$, (**d**–**f**) δ-$FAPbI_3$, and (**g**–**i**) $CsPbI_3$ single crystals.

Table S10. Source of the room-temperature experimental power factor ($S^2\sigma$) displayed in Figure 1d of the main text.

	$S^2\sigma$ (μW m^{-1} K^{-2})	Ref.
$MAPbI_3$ (MA = methylammonium)	433	[25]
$(4Tm)_2FASn_2I_7$ (4Tm = 3‴,4′-dimethyl[2,2′:5′,2″:5″,2‴-quaterthiophen]-5-yl)ethan-1-ammonium)	7.07	[26]
$CsSnI_3$	186	[27]
$CsSnI_{3-x}Cl_x$	150	[28]
PEDOT:Tos/Bi_2Te_3 (PEDOT = poly (3,4-ethylenedioxythiophene, Tos = tosylate)	1.35×10^3	[29]
poly(Ni-ett) (poly(nickel-ethylenetetrathiolate))	453	[30]

Table S11. Source of the room-temperature experimental TE figure of merit (zT) displayed in Figure 1h of the main text.

	zT	Ref.
$MASnI_3$	0.13	[25]
$CsSnI_3$	0.11	[27]
$CsSnI_{3-x}Cl_x$	0.10	[28]
PEDOT:Tos/Bi_2Te_3	0.58	[29]
poly(Ni-ett)	0.21	[30]

Section S3. Thermal Transport Properties

Table S12. Source of the room-temperature experimental thermal conductivity (κ_L) displayed in Figure 2b of the main text.

	κ_L (W m^{-1} K^{-1})			
	Single crystals	Polycrystals	Thin films	Ref.
$MAPbI_3$	0.50	0.30	–	[31]
	0.34	–	–	[32]
	0.34	–	0.33	[33]
	0.30	–	–	[34]
$MAPbBr_3$	0.51	–	–	[32]
	0.44	–	0.39	[33]
	0.37	–	–	[34]
$MAPbCl_3$	0.73	–	–	[32]
	0.50	–	0.50	[33]
	0.52	–	–	[34]
$FAPbBr_3$	0.49	–	–	[32]
$MASnI_3$	–	0.090	–	[25]
$(MA)_3Bi_2I_9$	–	0.23	–	[35]
$CsPbBr_3$	–	–	0.43	[36]
	–	0.46	–	[32]
$CsPb_2Br_5$	–	–	0.33	[25]

Table S13. Source of the room-temperature experimental elastic constant displayed in Figure 2c of the main text.

	Elastic constant (10^9 J m^{-3})	Ref.
$MAPbCl_3$ single crystal	23.0	[32]
$MAPbBr_3$ single crystal	17.8	[32]
$MAPbI_3$ single crystal	12.0	[32]
$FAPbBr_3$ single crystal	10.2	[32]
$CsPbBr_3$ single crystal	13.5	[32]

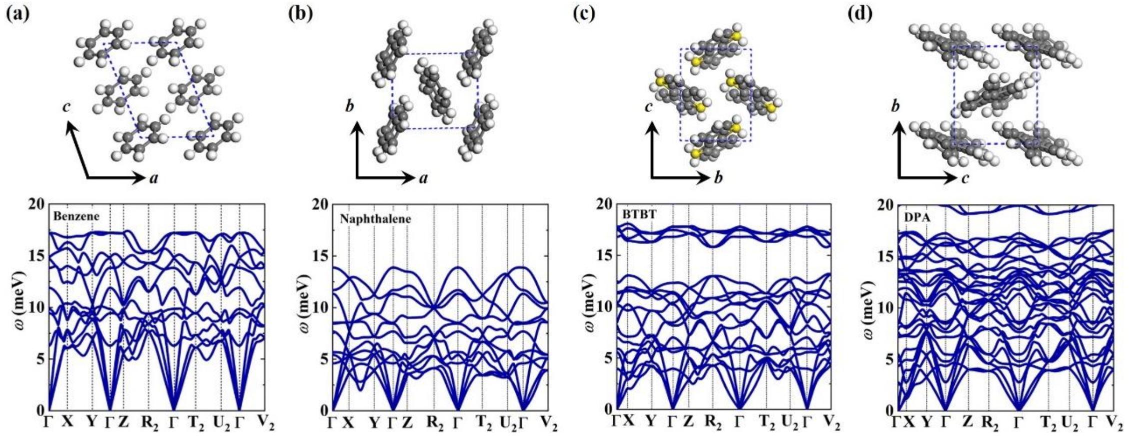

Figure S12. Crystalline structures and phonon distributions for four representative molecular crystals, including (**a**) benzene, (**b**) naphthalene, (**c**) [1]benzothieno[3,2-b]benzothiophene (BTBT), and (**d**) 2,6-diphenylanthracene (DPA). The blue dashed lines represent the crystal lattices. The carbon, sulphur, and hydrogen atoms are shown in grey, yellow, and white, respectively. The *k*-points of high symmetry in the first Brillouin zone are Γ = (0, 0, 0), X = (0.5, 0, 0), Y = (0, 0.5, 0), Z = (0, 0, 0.5), R_2 = (−0.5, −0.5, 0.5), T_2 = (0, −0.5, 0.5), U_2 = (−0.5, 0, 0.5), and V_2 = (0.5, −0.5, 0), respectively. Here, the phonon distributions are calculated by the finite displacement method[37] in VASP[4] and Phonopy[38]. The PAW method[1] with PBE functional[2] is utilized. The 2×2×2 supercells, and a *k*-mesh of 2×2×1 are used. The convergence criterion of the total energy is set to be 10^{-8} eV, and the cutoff energy for the plane-wave basis set is 500 eV.

Table S14. Group velocity of the longitudinal acoustic mode (v_L) along the *a*, *b*, and *c* axes, and heat capacity per volume at constant volume (c_V) for α-$FAPbI_3$, δ-$FAPbI_3$, and $CsPbI_3$ single crystals, respectively at room temperature.

	v_L (10^3 m s^{-1})			c_V (J cm^{-3} K^{-1})
	a	*b*	*c*	
α-$FAPbI_3$	3.00	3.01	4.40	0.94
δ-$FAPbI_3$	2.19	2.86	3.72	2.06
$CsPbI_3$	2.10	2.72	3.35	1.65

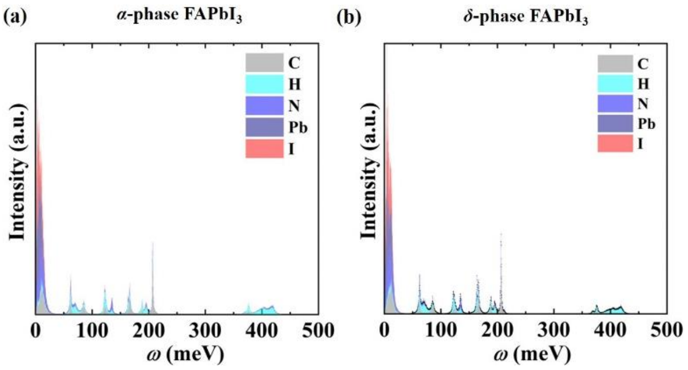

Figure S13. Vibrational DOS for (**a**) α-$FAPbI_3$ and (**b**) δ-$FAPbI_3$ single crystals, respectively at room temperature. The contributions of carbon, hydrogen, nitrogen, lead, and iodine atoms are displayed in gray, green, blue, dark blue, and red, respectively.

Table S15. Vibrational frequencies for the isolated $(CHN_2H_4)^+$. We carry out the calculations by using the B3LYP (Becke, 3-parameter, Lee-Yang-Parr) functional[39] and 6-31+G(d) basis set in Gaussian 16 program (version C.01).[40]

Number	ω (cm^{-1})

1	517.98
2	536.77
3	593.89
4	632.33
5	712.58
6	1044.7
7	1075.5
8	1130.8
9	1392.3
10	1420.3
11	1628.7
12	1712.4
13	1784.8
14	3238.6
15	3541.1
16	3555.1
17	3656.5
18	3657.5

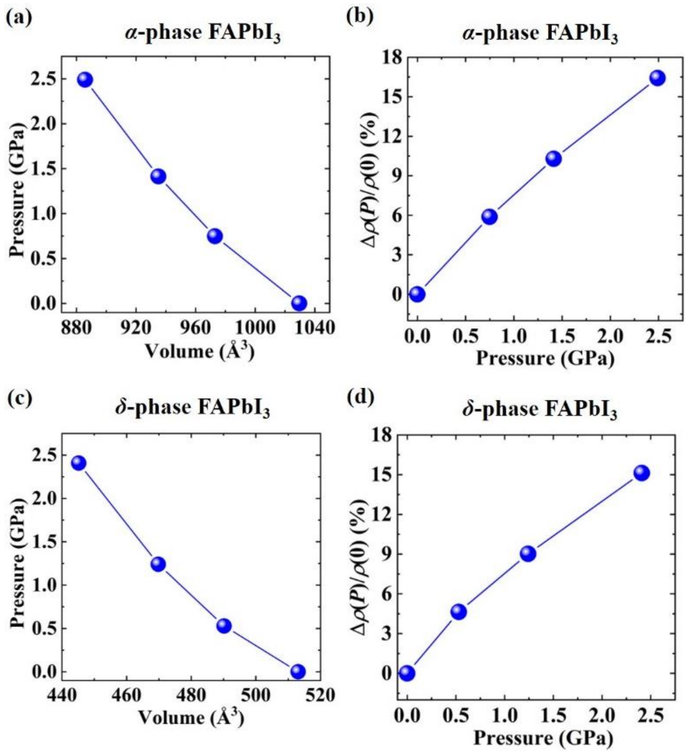

Figure S14. Relationship between the external pressure and the unit cell volume for (**a**) α-$FAPbI_3$ and (**c**) δ-$FAPbI_3$ single crystals, respectively. Relationships between the increasing ratio of the density $[\Delta\rho(P)/\rho(0)]$ and the external pressure for (**b**) α-$FAPbI_3$ and (**d**) δ-$FAPbI_3$ single crystals, respectively.

Section S4. Atomistic-Level Origin of Anharmonicity

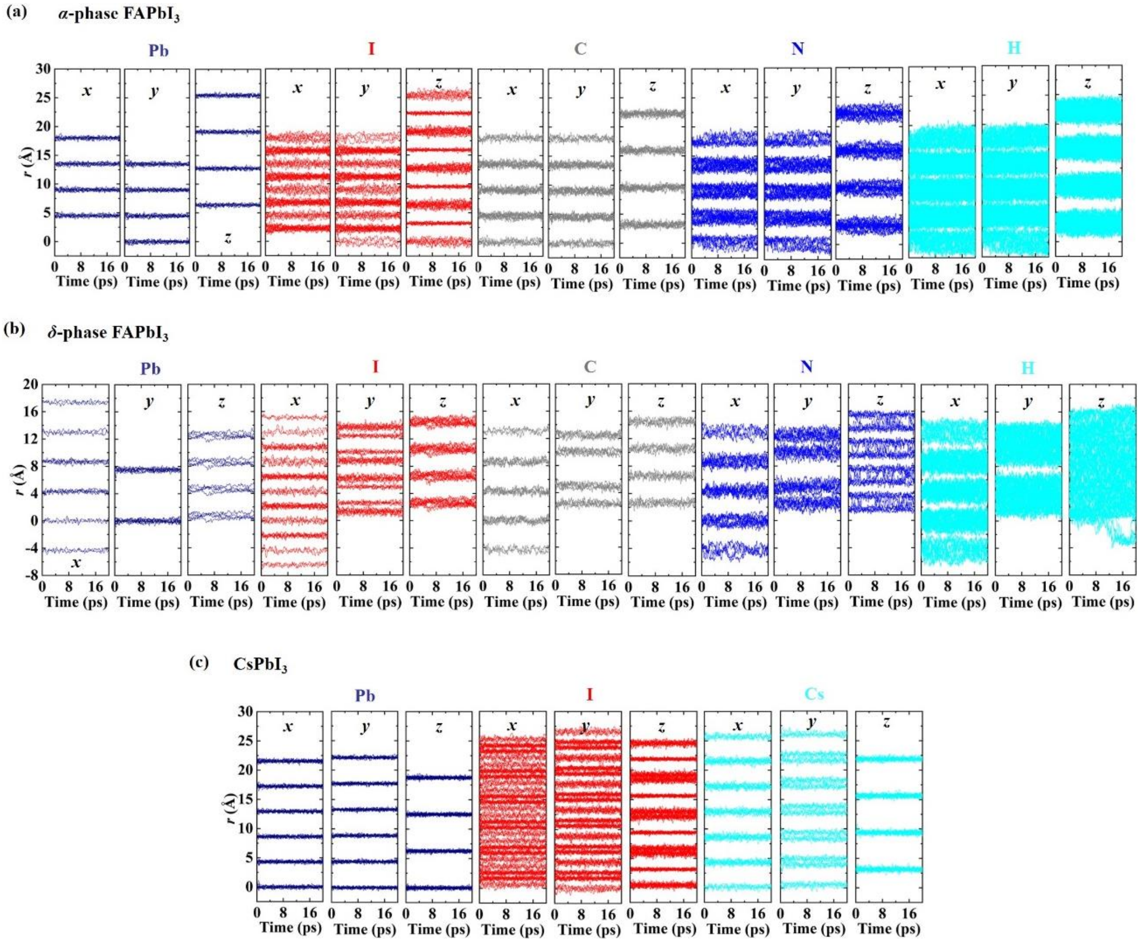

Figure S15. Atomic position ($\boldsymbol{r}$) evolution with time for (**a**) α-$FAPbI_3$, (**b**) δ-$FAPbI_3$, and (**c**) $CsPbI_3$ single crystals, respectively at room temperature based on the *ab initio* MD simulations.

Table S16. Standard deviation of position (Δr) for each element for crystalline α-$FAPbI_3$, δ-$FAPbI_3$, and $CsPbI_3$, respectively at room temperature based on the *ab initio* MD simulations. Here, $\Delta r_\alpha = \sqrt{\frac{1}{N}\sum_{i=1}^{N}(r_{\alpha i} - \overline{r_\alpha})^2}$, where N is the number of transient configurations; $r_{\alpha i}$ is the position of the i-th atom; $\overline{r_\alpha}$ is the average position of the specified atom for these transient configurations, and α is the cartesian direction. For the same element, the average standard deviations of position are present.

Δr (Å)	Pb			I			C			N			H		
	x	y	z	x	y	z	x	y	z	x	y	z	x	y	z
α-$FAPbI_3$	0.20	0.21	0.19	0.32	0.33	0.28	0.35	0.34	0.34	0.62	0.59	0.54	0.98	0.93	0.88
δ-$FAPbI_3$	0.21	0.23	0.28	0.24	0.26	0.34	0.33	0.34	0.33	0.52	0.53	0.74	0.91	0.92	1.0

Δr (Å)	Pb			I			Cs		
	x	y	z	x	y	z	x	y	z
$CsPbI_3$	0.17	0.16	0.17	0.24	0.24	0.23	0.29	0.29	0.24

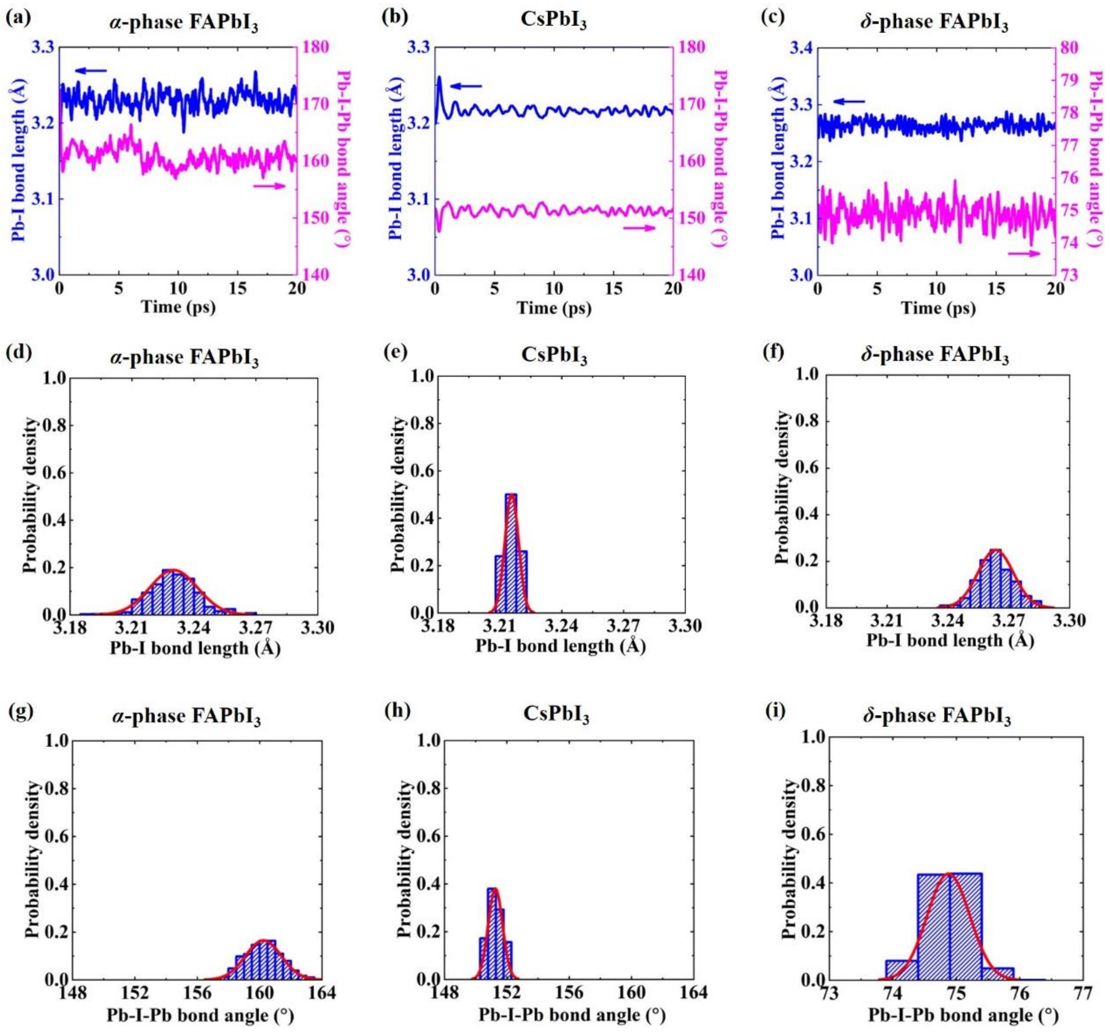

Figure S16. Pb−I bond length (blue) and Pb−I−Pb bond angle (pink) evolution with time for (**a**) α-FAPbI$_3$, (**b**) CsPbI$_3$, and (**c**) δ-FAPbI$_3$ single crystals, respectively based on the *ab initio* MD simulations at room temperature. Distribution of Pb−I bond length for (**d**) α-FAPbI$_3$, (**e**) CsPbI$_3$, and (**f**) δ-FAPbI$_3$ single crystals, respectively at room temperature. Distribution of Pb−I−Pb bond angle for (**g**) α-FAPbI$_3$, (**h**) CsPbI$_3$, and (**i**) δ-FAPbI$_3$ single crystals, respectively at room temperature. The red lines in (**d**−**i**) are the fitted Gaussian distributions.

Table S17. Average ($\langle d_{\mathrm{Pb-I}} \rangle$) and standard deviation ($\Delta d_{\mathrm{Pb-I}}$) of Pb−I bond lengths for crystalline α-FAPbI$_3$, δ-FAPbI$_3$, and CsPbI$_3$, respectively at room temperature based on the *ab initio* MD simulations. Average ($\langle \theta_{\mathrm{Pb-I-Pb}} \rangle$) and standard deviation ($\Delta \theta_{\mathrm{Pb-I-Pb}}$) of Pb−I−Pb bond angles for crystalline α-FAPbI$_3$, δ-FAPbI$_3$, and CsPbI$_3$, respectively at room temperature based on the *ab initio* MD simulations. The standard deviations normalized by the averages are also displayed in the table.

	$\langle d_{\mathrm{Pb-I}} \rangle$ (Å)	$\Delta d_{\mathrm{Pb-I}}$ (Å)	$\frac{\Delta d_{\mathrm{Pb-I}}}{\langle d_{\mathrm{Pb-I}} \rangle}$ (%)	$\langle \theta_{\mathrm{Pb-I-Pb}} \rangle$ (°)	$\Delta \theta_{\mathrm{Pb-I-Pb}}$ (°)	$\frac{\Delta \theta_{\mathrm{Pb-I-Pb}}}{\langle \theta_{\mathrm{Pb-I-Pb}} \rangle}$ (%)

α-$FAPbI_3$	3.23	0.012	0.37	160.2	1.16	0.72
δ-$FAPbI_3$	3.26	0.0088	0.27	74.9	0.34	0.45
$CsPbI_3$	3.22	0.003	0.09	151.3	0.46	0.30

Table S18. Charge of each atom in unit cell for α-$FAPbI_3$, δ-$FAPbI_3$, and $CsPbI_3$ single crystals, respectively. The average charges for the organic and inorganic components are also shown in the table.

α-$FAPbI_3$					
Atomic number	C	H	N	Pb	I
1	+1.28	+0.12	−1.29	+0.82	−0.54
2	+1.28	+0.12	−1.29	+0.82	−0.54
3	+1.28	+0.12	−1.29	+0.82	−0.54
4	+1.28	+0.13	−1.29	+0.82	−0.54
5	−	+0.47	−1.29	−	−0.56
6	−	+0.47	−1.29	−	−0.56
7	−	+0.47	−1.29	−	−0.56
8	−	+0.47	−1.29	−	−0.56
9	−	+0.47	−	−	−0.50
10	−	+0.47	−	−	−0.50
11	−	+0.47	−	−	−0.50
12	−	+0.47	−	−	−0.50
13	−	+0.51	−	−	−
14	−	+0.51	−	−	−
15	−	+0.51	−	−	−
16	−	+0.51	−	−	−
17	−	+0.51	−	−	−
18	−	+0.51	−	−	−
19	−	+0.51	−	−	−
20	−	+0.51	−	−	−
Average		$CHN_2H_4^+$		Pb	I
		+0.78		+0.82	−0.53

δ-$FAPbI_3$					
Atomic number	C	H	N	Pb	I
1	+1.23	+0.17	−1.30	+0.87	−0.55
2	+1.23	+0.17	−1.30	+0.87	−0.55
3	−	+0.50	−1.30	−	−0.52
4	−	+0.50	−1.30	−	−0.52
5	−	+0.48	−	−	−0.54
6	−	+0.48	−	−	−0.54
7	−	+0.49	−	−	−
8	−	+0.49	−	−	−
9	−	+0.48	−	−	−
10	−	+0.48	−	−	−
Average		$CHN_2H_4^+$		Pb	I
		+0.74		+0.87	−0.54

$CsPbI_3$			
Atomic number	Cs	Pb	I
1	+0.84	+0.84	−0.56
2	+0.84	+0.84	−0.56
3	+0.84	+0.84	−0.56
4	+0.84	+0.84	−0.56
5	−	−	−0.56
6	−	−	−0.56
7	−	−	−0.56

8	−	−	−0.56
9	−	−	−0.56
10	−	−	−0.56
11	−	−	−0.56
12	−	−	−0.56
Average	+0.84	+0.84	−0.56

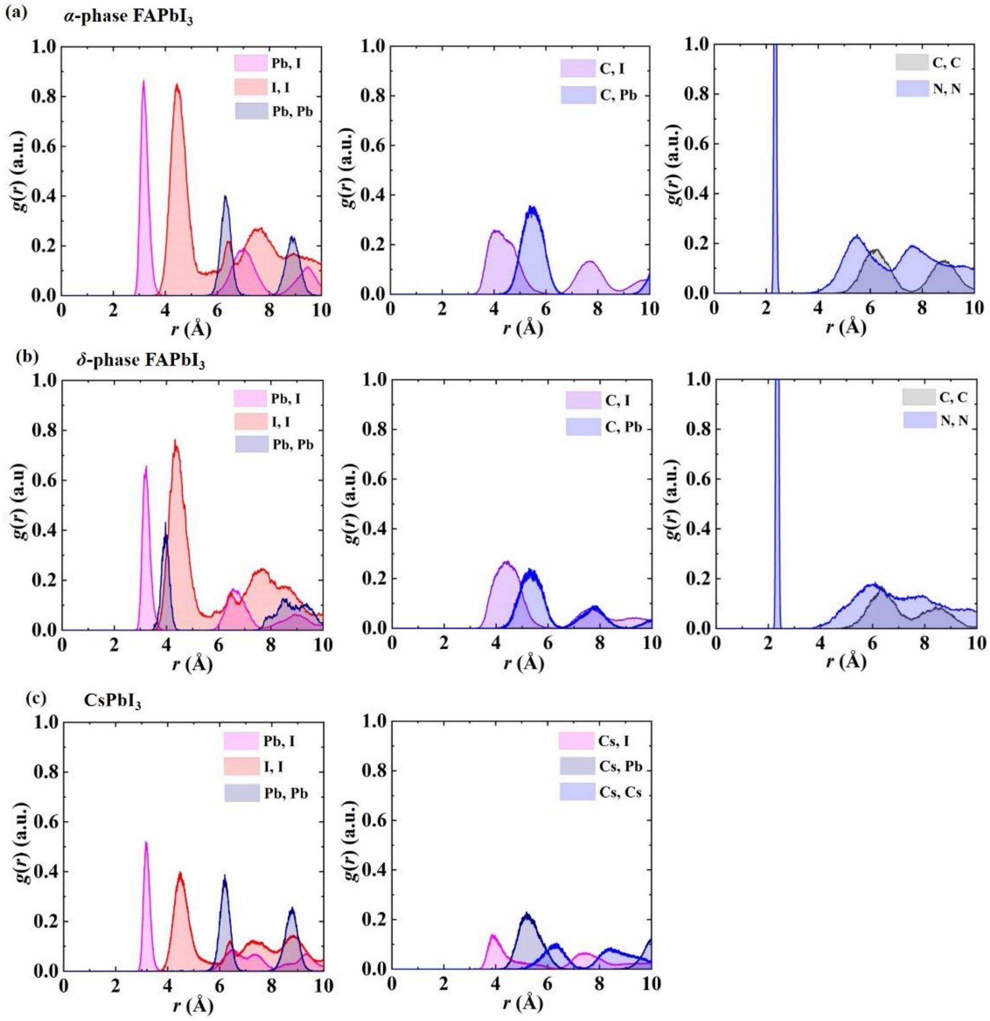

Figure S17. (**a**) Radial distribution function [$g(r)$] of Pb−I, I−I, Pb−Pb, C−I, C−Pb, C−C, and N−N for (**a**) α-$FAPbI_3$ and (**b**) δ-$FAPbI_3$ single crystals, respectively at room temperature based on the *ab initio* MD simulations. (**c**) Radial distribution function [$g(r)$] of Pb−I, I−I, Pb−Pb, Cs−I, Cs−Pb, and Cs−Cs for $CsPbI_3$ single crystals at room temperature based on the *ab initio* MD simulations.

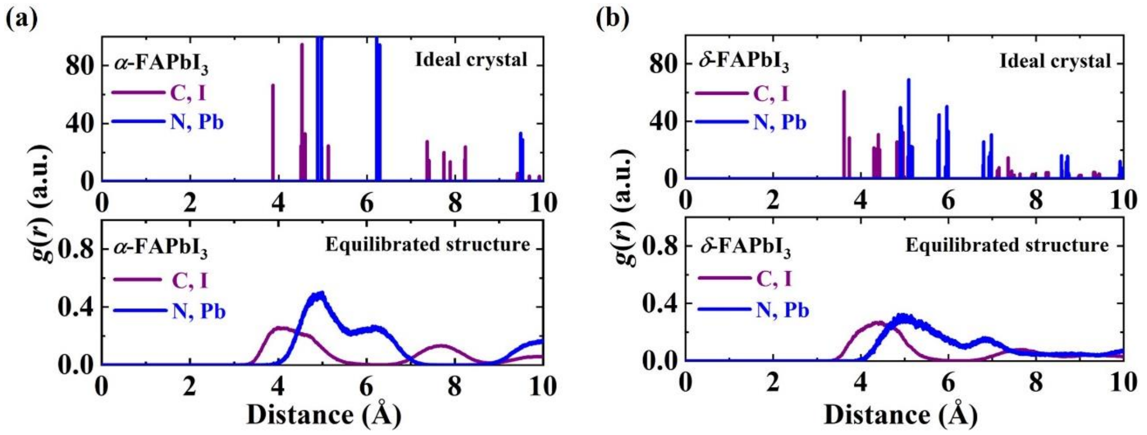

Figure S18. Radial distribution function $[g(r)]$ of thermally equilibrated structures at room temperature and that of ideal crystals for (**a**) α- and (**b**) δ-$FAPbI_3$, respectively. For crystalline α- and δ-$FAPbI_3$, our simulated radial distribution functions show that the distance distributions of C···I and N···Pb for the equilibrated structures markedly broaden, compared with those for the ideal crystals. Furthermore, combining our results of atomic charge analysis, we conclude that the electrostatic interaction networks between the inorganic frameworks and organic cations bring about the coupled motions of PbI^{3-} cage and $(CHN_2H_4)^+$, which leads to the broadened distance distribution in radial distribution functions.

Section S5. Charge Transport from the Standpoint of Electronic Structure and Lattice Dynamics

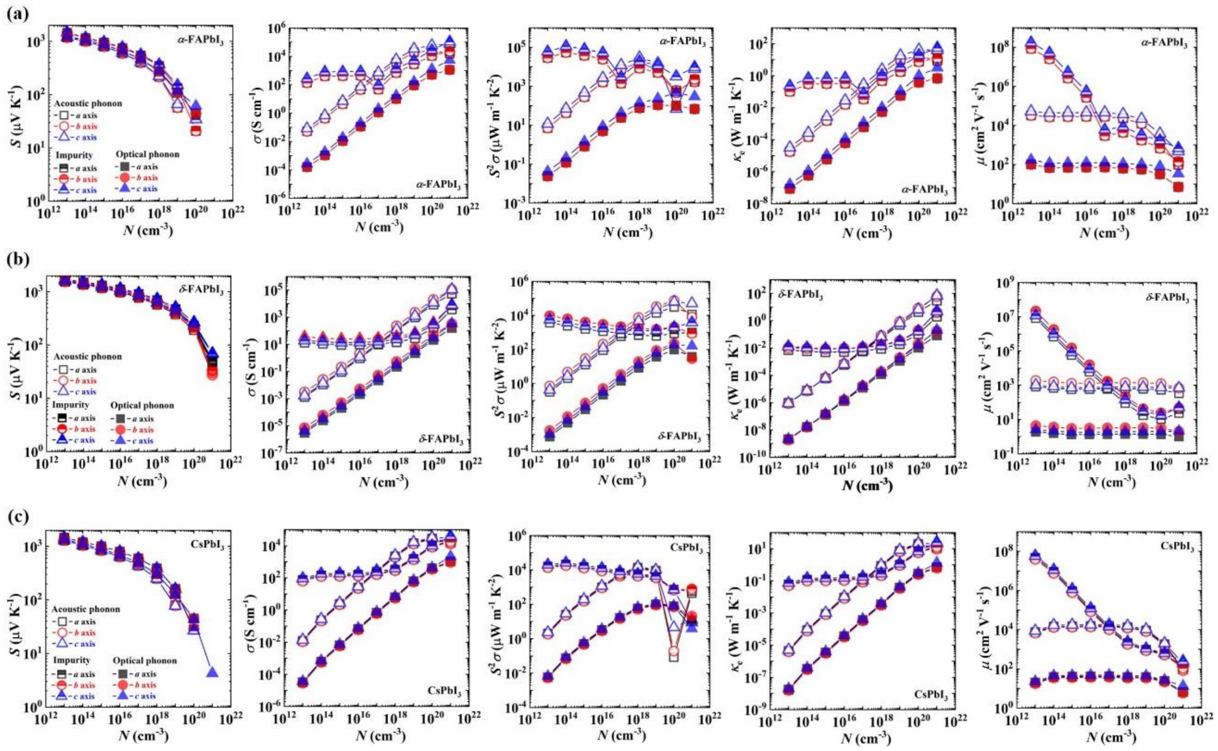

Figure S19. Room-temperature TE transport coefficients including Seebeck coefficient (S), conductivity (σ), power factor ($S^2\sigma$), electronic thermal conductivity (κ_e), and mobility (μ) along the *a* (black square), *b* (red circle) and *c* (blue triangle) axes contributed from acoustic phonon scattering, impurity scattering, and optical phonon scattering, respectively for (**a**) α-$FAPbI_3$, (**b**) δ-$FAPbI_3$, and (**c**) $CsPbI_3$ single crystals.

Table S19. Room-temperature TE transport coefficients including Seebeck coefficient (S), conductivity (σ), power factor ($S^2\sigma$), and electronic thermal conductivity (κ_e) along the *a*, *b* and *c* axes contributed from acoustic phonon scattering, impurity scattering, and optical phonon scattering, respectively for α-$FAPbI_3$ single crystal.

α-$FAPbI_3$, acoustic phonon scattering contribution												
N (cm^{-3})	S (μV K^{-1})			σ (S cm^{-1})			$S^2\sigma$ (μW m^{-1} K^{-2})			κ_e (W m^{-1} K^{-2})		
	a	*b*	*c*	*a*	*b*	*c*	*a*	*b*	*c*	*a*	*b*	*c*
10^{13}	1.18×10^{3}	1.18×10^{3}	1.18×10^{3}	0.0522	0.0521	0.0837	7.29	7.27	11.8	1.81×10^{-5}	1.82×10^{-5}	3.20×10^{-5}
10^{14}	999	999	1.00×10^{3}	0.434	0.434	0.696	43.4	43.2	70.3	1.52×10^{-4}	1.52×10^{-4}	2.66×10^{-4}
10^{15}	798	798	804	4.47	4.46	7.17	285	284	463	1.57×10^{-3}	1.57×10^{-3}	2.74×10^{-3}
10^{16}	597	597	603	45.8	45.8	73.5	1.64×10^{3}	1.63×10^{3}	2.68×10^{3}	0.0162	0.0162	0.0282
10^{17}	399	398	404	458	457	736	7.28×10^{3}	7.26×10^{3}	1.20×10^{4}	0.164	0.164	0.284
10^{18}	217	216	222	3.68×10^{3}	3.66×10^{3}	5.96×10^{3}	1.72×10^{4}	1.72×10^{4}	2.94×10^{4}	1.40	1.40	2.45
10^{19}	57.1	57.3	66.3	1.96×10^{4}	1.96×10^{4}	3.43×10^{4}	6.41×10^{3}	6.45×10^{3}	1.51×10^{4}	10.0	10.0	18.2
10^{20}	−15.7	−15.7	−3.39	2.46×10^{4}	2.46×10^{4}	6.04×10^{4}	606	607	69.4	17.0	17.0	42.0
10^{21}	−32.8	−32.8	−31.9	1.57×10^{4}	1.57×10^{4}	7.54×10^{4}	1.69×10^{3}	1.69×10^{3}	7.66×10^{3}	8.74	8.73	39.8
α-$FAPbI_3$, impurity scattering contribution												
N (cm^{-3})	S (μV K^{-1})			σ (S cm^{-1})			$S^2\sigma$ (μW m^{-1} K^{-2})			κ_e (W m^{-1} K^{-2})		
	a	*b*	*c*	*a*	*b*	*c*	*a*	*b*	*c*	*a*	*b*	*c*
10^{13}	1.45×10^{3}	1.45×10^{3}	1.46×10^{3}	138	138	275	2.89×10^{3}	2.8×10^{3}	5.92×10^{3}	0.104	0.104	0.232
10^{14}	1.15×10^{3}	1.15×10^{3}	1.17×10^{3}	410	409	818	5.46×10^{3}	5.46×10^{3}	1.13×10^{3}	0.310	0.310	0.689
10^{15}	953	954	972	422	422	842	3.84×10^{3}	3.84×10^{3}	7.97×10^{3}	0.319	0.319	0.709
10^{16}	752	753	771	438	437	874	2.48×10^{3}	2.48×10^{3}	5.20×10^{3}	0.331	0.330	0.735
10^{17}	545	545	564	48.0	47.9	95.8	1.42×10^{3}	1.42×10^{3}	3.05×10^{3}	0.0349	0.0349	0.0772
10^{18}	347	347	367	703	702	1.37×10^{3}	8.44×10^{3}	8.46×10^{3}	1.85×10^{3}	0.488	0.487	1.06
10^{19}	133	133	150	2.84×10^{3}	2.84×10^{3}	5.27×10^{3}	5.04×10^{3}	5.06×10^{3}	1.19×10^{4}	1.80	1.80	3.64

N (cm^{-3})	S (μV K^{-1}) *a*	*b*	*c*	σ (S cm^{-1}) *a*	*b*	*c*	$S^2\sigma$ (μW m^{-1} K^{-2}) *a*	*b*	*c*	κ_e (W m^{-1} K^{-2}) *a*	*b*	*c*
10^{20}	20.9	20.9	34.4	1.10×10^{4}	1.10×10^{4}	2.68×10^{4}	478	477	3.18×10^{3}	7.57	7.57	19.1
10^{21}	−31.8	−31.8	−29.6	2.24×10^{4}	2.24×10^{4}	1.09×10^{5}	2.27×10^{3}	2.27×10^{3}	9.56×10^{4}	12.2	12.2	56.2

α-$FAPbI_3$, optical phonon scattering contribution

N (cm^{-3})	S (μV K^{-1})			σ (S cm^{-1})			$S^2\sigma$ (μW m^{-1} K^{-2})			κ_e (W m^{-1} K^{-2})		
	a	*b*	*c*	*a*	*b*	*c*	*a*	*b*	*c*	*a*	*b*	*c*
10^{13}	1.22×10^{3}	1.22×10^{3}	1.23×10^{3}	1.61×10^{-4}	1.61×10^{-4}	2.76×10^{-4}	0.0242	0.0242	0.0418	8.64×10^{-8}	8.67×10^{-8}	1.59×10^{-7}
10^{14}	1.06×10^{3}	1.06×10^{3}	1.07×10^{3}	0.00109	0.00109	0.00186	0.122	0.122	0.211	5.82×10^{-7}	5.84×10^{-7}	1.07×10^{-6}
10^{15}	858	858	865	0.0112	0.0112	0.0192	0.824	0.824	1.43	5.99×10^{-6}	6.01×10^{-6}	1.10×10^{-5}
10^{16}	657	657	664	0.115	0.115	0.197	4.96	4.96	8.68	6.15×10^{-5}	6.17×10^{-5}	1.13×10^{-4}
10^{17}	458	458	466	1.16	1.16	1.99	24.4	24.4	43.1	6.23×10^{-4}	6.25×10^{-4}	1.15×10^{-3}
10^{18}	274	274	281	10.2	10.2	17.5	76.6	76.6	139	5.57×10^{-3}	5.58×10^{-3}	0.0103
10^{19}	109	109	118	91.3	91.3	166	108	108	231	0.0567	0.0567	0.110
10^{20}	44.3	44.3	61.3	513	513	1.28×10^{3}	101	100	479	0.384	0.383	1.01
10^{21}	−24.4	−24.4	−23.3	1.14×10^{3}	1.14×10^{3}	5.43×10^{3}	67.9	67.9	296	0.684	0.684	3.17

α-$FAPbI_3$, total

N (cm^{-3})	S (μV K^{-1})			σ (S cm^{-1})			$S^2\sigma$ (μW m^{-1} K^{-2})			κ_e (W m^{-1} K^{-2})		
	a	*b*	*c*	*a*	*b*	*c*	*a*	*b*	*c*	*a*	*b*	*c*
10^{13}	1.24×10^{3}	1.24×10^{3}	1.25×10^{3}	1.30×10^{-4}	1.30×10^{-4}	2.23×10^{-4}	0.0200	0.0201	0.0347	6.95×10^{-8}	6.97×10^{-8}	1.28×10^{-7}
10^{14}	1.06×10^{3}	1.06×10^{3}	1.06×10^{3}	1.08×10^{-3}	1.08×10^{-3}	1.85×10^{-3}	0.121	0.121	0.211	5.78×10^{-7}	5.80×10^{-7}	1.06×10^{-6}
10^{15}	858	858	865	0.0111	0.0112	0.0191	0.820	0.820	1.43	5.95×10^{-6}	5.97×10^{-6}	1.09×10^{-5}
10^{16}	657	657	664	0.114	0.114	0.196	4.94	4.94	8.64	6.11×10^{-5}	6.13×10^{-5}	1.12×10^{-4}
10^{17}	459	458	466	1.15	1.15	1.97	24.2	24.2	42.8	6.16×10^{-4}	6.18×10^{-4}	1.13×10^{-3}
10^{18}	276	276	283	9.92	9.91	17.1	75.4	75.3	137	5.42×10^{-3}	5.42×10^{-3}	0.0100
10^{19}	110	110	119	87.7	87.7	160	105	105	225	0.0542	0.0542	0.105
10^{20}	41.7	41.7	58.2	476	476	1.18×10^{3}	82.8	82.7	401	0.352	0.352	0.921
10^{21}	−25.6	−25.6	−24.6	1.01×10^{3}	1.01×10^{3}	4.83×10^{3}	66.4	66.5	291	0.600	0.600	2.77

Table S20. Room-temperature TE transport coefficients including Seebeck coefficient (S), conductivity (σ), power factor ($S^2\sigma$), and electronic thermal conductivity (κ_e) along the *a*, *b* and *c* axes contributed from acoustic phonon scattering, impurity scattering, and optical phonon scattering, respectively for δ-$FAPbI_3$ single crystal.

δ-$FAPbI_3$, acoustic phonon scattering contribution

N (cm^{-3})	S (μV K^{-1})			σ (S cm^{-1})			$S^2\sigma$ (μW m^{-1} K^{-2})			κ_e (W m^{-1} K^{-2})		
	a	*b*	*c*	*a*	*b*	*c*	*a*	*b*	*c*	*a*	*b*	*c*
10^{13}	1.54 $\times10^3$	1.55 $\times10^3$	1.58 $\times10^3$	1.34$\times10^{-3}$	3.02$\times10^{-3}$	1.66$\times10^{-3}$	0.317	0.723	0.416	9.52$\times10^{-7}$	8.66$\times10^{-7}$	8.84$\times10^{-7}$
10^{14}	1.36 $\times10^3$	1.36 $\times10^3$	1.40 $\times10^3$	0.0111	0.0251	0.0138	2.05	4.68	2.71	7.56$\times10^{-6}$	7.52$\times10^{-6}$	7.36$\times10^{-6}$
10^{15}	1.18 $\times10^3$	1.18 $\times10^3$	1.22 $\times10^3$	0.0927	0.209	0.115	12.8	29.2	17.0	6.00$\times10^{-5}$	6.52$\times10^{-5}$	6.13$\times10^{-5}$
10^{16}	974	982	1.02 $\times10^3$	0.954	2.15	1.18	90.6	207	122	5.83$\times10^{-4}$	7.01$\times10^{-4}$	6.31$\times10^{-4}$
10^{17}	773	781	815	9.82	22.1	12.2	587	1.35$\times10^3$	810	5.65$\times10^{-3}$	7.51$\times10^{-3}$	6.49$\times10^{-3}$
10^{18}	573	580	615	100	227	125	3.30$\times10^3$	7.64$\times10^3$	4.73$\times10^3$	0.0543	0.0801	0.0666
10^{19}	375	383	417	993	2.25$\times10^3$	1.25$\times10^3$	1.40$\times10^4$	3.29$\times10^4$	2.17$\times10^4$	0.506	0.829	0.667
10^{20}	190	194	228	8.64$\times10^3$	1.99$\times10^4$	1.19$\times10^4$	3.13$\times10^4$	7.50$\times10^4$	6.22$\times10^4$	4.40	8.03	6.52
10^{21}	46.0	27.3	67.2	5.43$\times10^4$	1.09$\times10^5$	1.13$\times10^5$	1.15$\times10^4$	8.12$\times10^3$	5.11$\times10^4$	29.1	58.8	69.0

δ-$FAPbI_3$, impurity scattering contribution

N (cm^{-3})	S (μV K^{-1})			σ (S cm^{-1})			$S^2\sigma$ (μW m^{-1} K^{-2})			κ_e (W m^{-1} K^{-2})		
	a	*b*	*c*	*a*	*b*	*c*	*a*	*b*	*c*	*a*	*b*	*c*
10^{13}	1.67 $\times10^3$	1.66 $\times10^3$	1.71 $\times10^3$	13.4	34.9	19.1	3.73$\times10^3$	9.60$\times10^3$	5.59$\times10^3$	7.08$\times10^{-3}$	0.0106	0.0128
10^{14}	1.48 $\times10^3$	1.48 $\times10^3$	1.53 $\times10^3$	11.2	29.1	15.9	2.46$\times10^3$	6.33$\times10^3$	3.71$\times10^3$	5.92$\times10^{-3}$	9.45$\times10^{-3}$	0.0106
10^{15}	1.30 $\times10^3$	1.29 $\times10^3$	1.34 $\times10^3$	9.30	24.2	13.2	1.58$\times10^3$	4.05$\times10^3$	2.40$\times10^3$	4.95$\times10^{-3}$	8.40$\times10^{-3}$	8.85$\times10^{-3}$
10^{16}	1.10 $\times10^3$	1.09 $\times10^3$	1.14 $\times10^3$	9.67	25.2	13.8	1.17$\times10^3$	3.00$\times10^3$	1.81$\times10^3$	5.20$\times10^{-3}$	9.35$\times10^{-3}$	9.24$\times10^{-3}$
10^{17}	899	891	940	10.7	27.8	16.0	868	2.20$\times10^3$	1.42$\times10^3$	6.00$\times10^{-3}$	0.0112	0.0106
10^{18}	691	687	729	14.4	36.5	23.8	690	1.72$\times10^3$	1.27$\times10^3$	0.00885	0.0164	0.0152
10^{19}	468	472	506	28.4	68.7	50.4	624	1.53$\times10^3$	1.29$\times10^3$	0.0193	0.0340	0.0310
10^{20}	228	232	267	169	392	286	876	2.11$\times10^3$	2.03$\times10^3$	0.103	0.193	0.168
10^{21}	51.4	33.6	69.3	3.76$\times10^3$	7.63$\times10^3$	8.04$\times10^3$	995	862	3.86$\times10^3$	2.00	4.06	4.80

δ-$FAPbI_3$, optical phonon scattering contribution

N (cm^{-3})	S (μV K^{-1})			σ (S cm^{-1})			$S^2\sigma$ (μW m^{-1} K^{-2})			κ_e (W m^{-1} K^{-2})		
	a	*b*	*c*	*a*	*b*	*c*	*a*	*b*	*c*	*a*	*b*	*c*
10^{13}	1.57 $\times10^3$	1.57 $\times10^3$	1.60 $\times10^3$	3.04$\times10^{-6}$	7.04$\times10^{-6}$	4.12$\times10^{-6}$	7.49$\times10^{-4}$	1.74$\times10^{-3}$	1.06$\times10^{-3}$	2.11$\times10^{-9}$	1.88$\times10^{-9}$	2.16$\times10^{-9}$
10^{14}	1.39 $\times10^3$	1.39 $\times10^3$	1.42 $\times10^3$	2.53$\times10^{-5}$	5.86$\times10^{-5}$	3.43$\times10^{-5}$	4.87$\times10^{-3}$	0.0113	6.95$\times10^{-3}$	1.68$\times10^{-8}$	1.65$\times10^{-8}$	1.80$\times10^{-8}$
10^{15}	1.20 $\times10^3$	1.21 $\times10^3$	1.24 $\times10^3$	2.11$\times10^{-4}$	4.88$\times10^{-4}$	2.86$\times10^{-4}$	0.0306	0.0712	0.0440	1.34$\times10^{-7}$	1.44$\times10^{-7}$	1.50$\times10^{-7}$
10^{16}	1.00 $\times10^3$	1.01 $\times10^3$	1.04 $\times10^3$	2.17$\times10^{-3}$	5.02$\times10^{-3}$	2.94$\times10^{-3}$	0.218	0.509	0.318	1.31$\times10^{-6}$	1.57$\times10^{-6}$	1.54$\times10^{-6}$
10^{17}	802	806	839	0.0223	0.0516	0.0302	1.44	3.36	2.13	1.28$\times10^{-5}$	1.69$\times10^{-5}$	1.58$\times10^{-5}$

10^{18}	602	606	638	0.229	0.530	0.310	8.29	19.4	12.6	1.24×10^{-4}	1.82×10^{-4}	1.63×10^{-4}
10^{19}	404	408	440	2.28	5.29	3.12	37.3	87.9	60.4	1.17×10^{-3}	1.91×10^{-3}	1.64×10^{-3}
10^{20}	213	213	245	21.6	50.5	31.5	97.9	229	190	0.0106	0.0195	0.0167
10^{21}	48.1	29.7	69.1	162	324	339	37.5	28.6	162	0.0849	0.171	0.206

δ-$FAPbI_3$, total												
N (cm^{-3})	S (μV K^{-1})			σ (S cm^{-1})			$S^2\sigma$ (μW m^{-1} K^{-2})			κ_e (W m^{-1} K^{-2})		
	a	*b*	*c*	*a*	*b*	*c*	*a*	*b*	*c*	*a*	*b*	*c*
10^{13}	1.57×10^3	1.57×10^3	1.60×10^3	3.03×10^{-6}	7.02×10^{-6}	4.11×10^{-6}	7.47×10^{-4}	1.74×10^{-3}	1.06×10^{-3}	2.10×10^{-9}	1.88×10^{-9}	2.15×10^{-9}
10^{14}	1.39×10^3	1.39×10^3	1.42×10^3	2.53×10^{-5}	5.85×10^{-5}	3.42×10^{-5}	4.86×10^{-3}	0.0113	6.94×10^{-3}	1.68×10^{-8}	1.65×10^{-8}	1.79×10^{-8}
10^{15}	1.20×10^3	1.21×10^3	1.24×10^3	2.10×10^{-4}	4.87×10^{-4}	2.85×10^{-4}	0.0305	0.0710	0.0439	1.34×10^{-7}	1.44×10^{-7}	1.49×10^{-7}
10^{16}	1.00×10^3	1.01×10^3	1.04×10^3	2.16×10^{-3}	5.00×10^{-3}	2.93×10^{-3}	0.218	0.508	0.317	1.31×10^{-6}	1.56×10^{-6}	1.53×10^{-6}
10^{17}	803	807	839	0.0221	0.0513	0.0300	1.43	3.34	2.12	1.26×10^{-5}	1.68×10^{-5}	1.57×10^{-5}
10^{18}	604	608	640	0.221	0.514	0.302	8.07	19.0	12.4	1.19×10^{-4}	1.76×10^{-4}	1.58×10^{-4}
10^{19}	409	412	444	2.06	4.78	2.88	34.4	81.2	56.8	1.07×10^{-3}	1.76×10^{-3}	1.52×10^{-3}
10^{20}	214	215	247	19.0	44.4	28.2	87.3	205	173	9.52×10^{-3}	0.0176	0.0151
10^{21}	48.2	29.8	69.1	155	310	324	36.0	27.6	155	0.0812	0.164	0.197

Table S21. Room-temperature TE transport coefficients including Seebeck coefficient (S), conductivity (σ), power factor ($S^2\sigma$), and electronic thermal conductivity (κ_e) along the *a*, *b* and *c* axes contributed from acoustic phonon scattering, impurity scattering, and optical phonon scattering, respectively for $CsPbI_3$ single crystal.

$CsPbI_3$, acoustic phonon scattering contribution												
N (cm^{-3})	S (μV K^{-1})			σ (S cm^{-1})			$S^2\sigma$ (μW m^{-1} K^{-2})			κ_e (W m^{-1} K^{-2})		
	a	*b*	*c*	*a*	*b*	*c*	*a*	*b*	*c*	*a*	*b*	*c*
10^{13}	1.30×10^3	1.30×10^3	1.30×10^3	0.0120	0.0109	0.0140	2.01	1.83	2.34	4.41×10^{-6}	4.00×10^{-6}	5.08×10^{-6}
10^{14}	1.04×10^3	1.04×10^3	1.04×10^3	0.232	0.212	0.271	25.1	22.9	29.3	8.60×10^{-5}	7.80×10^{-5}	9.90×10^{-5}
10^{15}	839	839	838	23.9	21.8	27.9	168	154	196	8.87×10^{-4}	8.04×10^{-4}	1.02×10^{-3}
10^{16}	639	638	638	24.5	22.4	28.6	1.00×10^3	913	1.16×10^3	9.13×10^{-3}	8.28×10^{-3}	0.01.05
10^{17}	439	439	438	248	226	289	4.78×10^3	4.36×10^3	5.56×10^3	0.0929	0.0842	0.107
10^{18}	251	251	250	2.15×10^3	1.97×10^3	2.51×10^3	1.36×10^3	1.24×10^3	1.58×10^3	0.845	0.764	0.971
10^{19}	79.7	78.6	78.0	1.44×10^4	1.31×10^4	1.67×10^4	9.19×10^3	8.10×10^3	1.02×10^4	7.23	6.54	8.31
10^{2}	−0.1	−0.2	−1.1	2.86×	2.57×	3.22×	0.084	0.193	4.57	19.4	17.4	21.8

0	72	74	9	10^4	10^4	10^4	7					
10^{21}	−17.2	−21.8	−2.02	1.53×10^4	1.34×10^4	3.08×10^4	453	640	12.6	10.9	9.56	19.8

$CsPbI_3$, impurity scattering contribution

N (cm^{-3})	S (μV K^{-1})			σ (S cm^{-1})			$S^2\sigma$ (μW m^{-1} K^{-2})			κ_e (W m^{-1} K^{-2})		
	a	*b*	*c*	*a*	*b*	*c*	*a*	*b*	*c*	*a*	*b*	*c*
10^{13}	1.46×10^3	1.46×10^3	1.46×10^3	74.9	63.5	93.1	1.60×10^4	1.35×10^4	1.98×10^4	0.0599	0.0507	0.0735
10^{14}	1.21×10^3	1.20×10^3	1.20×10^3	146	123	181	2.12×10^4	1.79×10^4	2.63×10^4	0.117	0.0986	0.143
10^{15}	1.00×10^3	1.00×10^3	1.00×10^3	150	127	187	1.52×10^4	1.28×10^4	1.88×10^4	0.120	0.102	0.147
10^{16}	804	801	802	157	134	196	1.02×10^4	8.58×10^3	1.26×10^4	0.126	0.107	0.154
10^{17}	599	596	598	187	161	232	6.72×10^3	5.74×10^3	8.28×10^3	0.147	0.126	0.180
10^{18}	389	386	388	325	288	387	4.92×10^3	4.28×10^3	5.84×10^3	0.241	0.212	0.284
10^{19}	161	159	160	1.52×10^3	1.38×10^3	1.73×10^3	3.94×10^3	3.94×10^3	4.44×10^3	1.02	0.922	1.16
10^{20}	28.4	28.4	26.7	9.18×10^3	8.38×10^3	1.02×10^4	739	676	726	6.19	5.64	6.89
10^{21}	−17.2	−21.4	−1.48	2.01×10^4	1.74×10^4	3.98×10^4	593	796	8.73	14.1	12.2	25.4

$CsPbI_3$, optical phonon scattering contribution

N (cm^{-3})	S (μV K^{-1})			σ (S cm^{-1})			$S^2\sigma$ (μW m^{-1} K^{-2})			κ_e (W m^{-1} K^{-2})		
	a	*b*	*c*	*a*	*b*	*c*	*a*	*b*	*c*	*a*	*b*	*c*
10^{13}	1.35×10^3	1.35×10^3	1.35×10^3	3.20×10^{-5}	2.89×10^{-5}	3.68×10^{-5}	5.85×10^{-3}	5.28×10^{-3}	6.72×10^{-3}	1.75×10^{-8}	1.58×10^{-8}	2.00×10^{-8}
10^{14}	1.10×10^3	1.10×10^3	1.10×10^3	6.22×10^{-4}	5.62×10^{-4}	7.12×10^{-4}	0.0748	0.0674	0.0859	3.40×10^{-7}	3.07×10^{-7}	3.89×10^{-7}
10^{15}	895	895	895	6.40×10^{-3}	5.78×10^{-3}	7.36×10^{-3}	0.513	0.463	0.589	3.50×10^{-6}	3.16×10^{-6}	4.00×10^{-6}
10^{16}	695	694	694	0.0658	0.0594	0.0757	3.17	2.86	3.64	3.59×10^{-5}	3.25×10^{-5}	4.12×10^{-5}
10^{17}	495	494	494	0.669	0.604	0.769	16.4	14.8	18.8	3.66×10^{-4}	3.31×10^{-4}	4.19×10^{-4}
10^{18}	306	306	306	6.15	5.55	7.07	57.6	51.8	66.0	3.41×10^{-3}	3.08×10^{-3}	3.90×10^{-3}
10^{19}	128	128	127	61.5	55.3	70.4	101	90.3	114	0.0377	0.0339	0.0430
10^{20}	44.9	44.9	43.7	388	348	435	78.2	70.3	83.0	0.278	0.250	0.313
10^{21}	−10.5	−14.9	4.27	1.06×10^3	940	2.13×10^3	11.6	20.9	3.88	0.708	0.624	1.30

$CsPbI_3$, total

N (cm^{-3})	S (μV K^{-1})			σ (S cm^{-1})			$S^2\sigma$ (μW m^{-1} K^{-2})			κ_e (W m^{-1} K^{-2})		
	a	*b*	*c*	*a*	*b*	*c*	*a*	*b*	*c*	*a*	*b*	*c*
10^{13}	1.35×10^3	1.35×10^3	1.35×10^3	3.19×10^{-5}	2.88×10^{-5}	3.67×10^{-5}	5.85×10^{-3}	5.26×10^{-3}	6.70×10^{-3}	1.74×10^{-8}	1.57×10^{-8}	1.99×10^{-8}
10^{14}	1.10×10^3	1.10×10^3	1.10×10^3	6.20×10^{-4}	5.60×10^{-4}	7.13×10^{-4}	0.0745	0.0672	0.0856	3.38×10^{-7}	3.05×10^{-7}	3.87×10^{-7}
10^{15}	895	894	894	6.38×10^{-3}	5.76×10^{-3}	7.34×10^{-3}	0.511	0.461	0.587	3.47×10^{-6}	3.14×10^{-6}	3.98×10^{-6}

10^{16}	694	694	694	0.0655	0.0591	0.0753	3.16	2.85	3.63	3.57×10^{-5}	3.23×10^{-5}	4.09×10^{-5}
10^{17}	496	495	495	0.662	0.597	0.761	16.2	14.6	18.6	3.61×10^{-4}	3.26×10^{-4}	4.13×10^{-4}
10^{18}	308	308	307	5.96	5.37	6.85	56.5	50.8	64.8	3.30×10^{-3}	2.98×10^{-3}	3.78×10^{-3}
10^{19}	129	129	128	58.7	52.8	67.2	97.8	87.6	111	0.0359	0.0323	0.0410
10^{20}	43.4	43.5	42.2	367	329	411	69.2	62.2	73.2	0.261	0.234	0.293
10^{21}	−11.0	−15.5	3.82	948	838	1.90×10^{3}	11.6	20.1	2.77	0.634	0.558	1.17

Table S22. Room-temperature hole mobility (μ) along the *a*, *b* and *c* axes contributed from acoustic phonon scattering, impurity scattering, and optical phonon scattering for α-$FAPbI_3$, δ-$FAPbI_3$, and $CsPbI_3$ single crystals, respectively.

N (cm^{-3})	μ (cm^2 V^{-1} s^{-1}), α-$FAPbI_3$											
	Acoustic phonon			Impurity			Optical phonon			Total		
	a	*b*	*c*	*a*	*b*	*c*	*a*	*b*	*c*	*a*	*b*	*c*
10^{13}	3.26×10^{4}	3.25×10^{4}	5.22×10^{4}	8.62×10^{7}	8.60×10^{7}	1.70×10^{8}	101	101	172	81.2	81.3	139
10^{14}	2.71×10^{4}	2.71×10^{4}	4.35×10^{4}	2.56×10^{7}	2.56×10^{7}	5.11×10^{7}	67.8	67.9	116	67.6	67.6	116
10^{15}	2.79×10^{4}	2.78×10^{4}	4.47×10^{4}	2.64×10^{6}	2.63×10^{6}	5.26×10^{6}	69.8	69.8	120	69.6	69.6	119
10^{16}	2.86×10^{4}	2.86×10^{4}	4.59×10^{4}	2.73×10^{5}	2.73×10^{5}	5.46×10^{5}	71.7	71.8	123	71.4	71.5	122
10^{17}	2.86×10^{4}	2.85×10^{4}	4.59×10^{4}	3.00×10^{3}	3.00×10^{3}	5.98×10^{3}	72.4	72.5	124	71.8	71.9	123
10^{18}	2.30×10^{4}	2.29×10^{4}	3.72×10^{4}	4.38×10^{3}	4.38×10^{3}	8.57×10^{3}	63.5	63.5	110	61.9	61.9	107
10^{19}	1.23×10^{4}	1.22×10^{4}	2.14×10^{4}	1.78×10^{3}	1.78×10^{3}	3.29×10^{3}	57.0	57.0	104	54.8	54.7	99.6
10^{20}	1.54×10^{3}	1.54×10^{3}	3.77×10^{3}	684	684	1.68×10^{3}	32.0	32.0	79.7	29.7	29.7	73.8
10^{21}	98.2	98.2	471	140	140	680	7.09	7.09	33.9	6.31	6.31	30.1

N (cm^{-3})	μ (cm^2 V^{-1} s^{-1}), δ-$FAPbI_3$											
	Acoustic phonon			Impurity			Optical phonon			Total		
	a	*b*	*c*	*a*	*b*	*c*	*a*	*b*	*c*	*a*	*b*	*c*
10^{13}	835	1.88×10^{3}	1.04×10^{3}	8.37×10^{6}	2.18×10^{7}	1.19×10^{7}	1.90	4.39	2.57	1.89	4.38	2.57
10^{14}	695	1.57×10^{3}	863	6.97×10^{5}	1.82×10^{6}	9.93×10^{5}	1.58	3.66	2.14	1.58	3.64	2.14
10^{15}	579	1.30×10^{3}	719	5.81×10^{4}	1.51×10^{5}	8.28×10^{4}	1.32	3.04	1.78	1.31	3.04	1.78
10^{16}	596	1.34×10^{3}	740	6.04×10^{3}	1.57×10^{4}	8.65×10^{3}	1.35	3.13	1.84	1.35	3.12	1.83
10^{17}	613	1.38×10^{3}	761	671	1.73×10^{3}	1.00×10^{3}	1.39	3.22	1.89	1.38	3.20	1.87
10^{18}	628	1.42×10^{3}	781	90.1	228	149	1.43	3.31	1.94	1.38	3.21	1.88

10^{19}	620	1.40×10^{3}	779	17.8	42.9	31.5	1.42	3.30	1.95	1.28	2.98	1.80
10^{20}	539	1.24×10^{3}	744	10.5	24.5	17.8	1.34	3.15	1.97	1.18	2.77	1.76
10^{21}	339	680	707	23.5	47.6	50.2	1.01	2.02	2.11	0.965	1.94	2.02

N (cm^{-3})	μ (cm^2 V^{-1} s^{-1}), $CsPbI_3$											
	Acoustic phonon			Impurity			Optical phonon			Total		
	a	*b*	*c*	*a*	*b*	*c*	*a*	*b*	*c*	*a*	*b*	*c*
10^{13}	7.46×10^{3}	6.81×10^{3}	8.71×10^{3}	4.67×10^{7}	3.96×10^{7}	5.81×10^{7}	20.0	18.0	23.0	19.9	18.0	22.9
10^{14}	1.45×10^{4}	1.32×10^{4}	1.69×10^{4}	9.08×10^{6}	7.70×10^{6}	1.13×10^{7}	38.8	35.1	44.7	38.7	35.0	44.5
10^{15}	1.49×10^{4}	1.36×10^{4}	1.74×10^{4}	9.37×10^{5}	7.94×10^{5}	1.16×10^{6}	40.0	36.1	46.0	39.8	36.0	45.8
10^{16}	1.53×10^{4}	1.40×10^{4}	1.79×10^{4}	9.82×10^{5}	8.34×10^{5}	1.22×10^{5}	41.1	37.1	47.2	40.9	36.9	47.0
10^{17}	1.55×10^{4}	1.41×10^{4}	1.80×10^{4}	1.17×10^{4}	1.01×10^{4}	1.45×10^{4}	41.8	37.7	48.0	41.3	37.3	47.5
10^{18}	1.34×10^{4}	1.23×10^{4}	1.57×10^{4}	2.03×10^{3}	1.80×10^{3}	2.42×10^{3}	38.4	34.6	44.1	37.2	33.6	42.8
10^{19}	9.02×10^{3}	8.19×10^{3}	1.04×10^{4}	951	861	1.08×10^{3}	38.4	34.5	44.0	36.6	33.0	42.0
10^{20}	1.79×10^{3}	1.60×10^{3}	2.01×10^{3}	573	523	634	24.2	21.7	27.1	22.9	20.6	25.6
10^{21}	95.3	83.8	192	125	109	248	6.64	5.87	13.3	5.92	5.23	11.8

Table S23. Average hole relaxation time ($\langle\tau_{\mathrm{acoustic}}\rangle$) and *a*-, *b*-, and *c*-axis hole mean free path ($\langle l_{\mathrm{acoustic}}\rangle$) contributed from the acoustic phonon scattering for α-$FAPbI_3$, δ-$FAPbI_3$, and $CsPbI_3$ single crystals, respectively at room temperature. Here, $\langle\tau\rangle = \frac{\sum_k \tau_k[1-f_0(E_k,E_{\mathrm{F}},T)]}{\sum_k[1-f_0(E_k,E_{\mathrm{F}},T)]}$, and $\langle \boldsymbol{l}\rangle = \frac{\sum_k \boldsymbol{v}_k\tau_k[1-f_0(E_k,E_{\mathrm{F}},T)]}{\sum_k[1-f_0(E_k,E_{\mathrm{F}},T)]}$.

	$\langle\tau_{\mathrm{acoustic}}\rangle$ (fs)	$\langle l_{\mathrm{acoustic}}\rangle$ (Å)		
		a axis	*b* axis	*c* axis
α-$FAPbI_3$	84.0	98	98	446
δ-$FAPbI_3$	2.44×10^{3}	3.02×10^{3}	3.65×10^{3}	1.40×10^{4}
$CsPbI_3$	296	888	485	1.00×10^{3}

Table S24. Average hole relaxation time ($\langle\tau_{\mathrm{optical}}\rangle$) and *a*-, *b*-, and *c*-axis hole mean free path ($\langle l_{\mathrm{optical}}\rangle$) contributed from the optical phonon scattering for α-$FAPbI_3$, δ-$FAPbI_3$, and $CsPbI_3$ single crystals, respectively at room temperature.

	$\langle\tau_{\mathrm{optical}}\rangle$ (fs)	$\langle l_{\mathrm{optical}}\rangle$ (Å)		
		a axis	*b* axis	*c* axis
α-$FAPbI_3$	8.81	11.4	11.4	54.5
δ-$FAPbI_3$	2.00	3.00	3.60	14.4
$CsPbI_3$	5.64	21.1	13.8	24.3

Table S25. Average hole relaxation time ($\langle\tau_{\text{impurity}}\rangle$) and *a*-, *b*-, and *c*-axis hole mean free path ($\langle l_{\text{impurity}}\rangle$) contributed from the charged impurity scattering for α-$FAPbI_3$, δ-$FAPbI_3$, and $CsPbI_3$ single crystals, respectively at room temperature.

α-$FAPbI_3$				
N (cm^{-3})	$<\tau_{impurity}>$ (fs)	$<l_{impurity}>$ (Å)		
		a axis	*b* axis	*c* axis
10^{13}	2.10×10^{7}	4.67×10^{7}	4.67×10^{7}	2.06×10^{8}
10^{14}	2.10×10^{6}	4.67×10^{6}	4.67×10^{6}	2.06×10^{7}
10^{15}	2.10×10^{5}	4.67×10^{5}	4.67×10^{5}	2.06×10^{6}
10^{16}	2.12×10^{4}	4.70×10^{4}	4.70×10^{4}	2.08×10^{5}
10^{17}	2.23×10^{3}	4.92×10^{3}	4.92×10^{3}	2.17×10^{4}
10^{18}	283	614	614	2.68×10^{3}
10^{19}	55.1	113	113	462
10^{20}	33.3	53.1	53.1	216
10^{21}	92.2	116	116	513
δ-$FAPbI_3$				
N (cm^{-3})	$<\tau_{impurity}>$ (fs)	$<l_{impurity}>$ (Å)		
		a axis	*b* axis	*c* axis
10^{13}	3.12×10^{6}	5.47×10^{6}	6.87×10^{6}	3.40×10^{7}
10^{14}	3.12×10^{5}	5.47×10^{5}	6.87×10^{5}	3.40×10^{6}
10^{15}	3.13×10^{4}	5.48×10^{4}	6.88×10^{4}	3.40×10^{5}
10^{16}	3.15×10^{3}	5.52×10^{3}	6.92×10^{3}	3.43×10^{4}
10^{17}	337	586	732	3.60×10^{3}
10^{18}	46.6	82.4	102	455
10^{19}	17.1	30.5	37.2	130
10^{20}	27.2	40.4	48.5	170
10^{21}	137	177	213	798
$CsPbI_3$				
N (cm^{-3})	$<\tau_{impurity}>$ (fs)	$<l_{impurity}>$ (Å)		
		a axis	*b* axis	*c* axis
10^{13}	1.09×10^{7}	6.81×10^{7}	3.77×10^{7}	7.80×10^{7}
10^{14}	1.09×10^{6}	6.81×10^{6}	3.77×10^{6}	7.80×10^{6}
10^{15}	1.10×10^{5}	6.82×10^{5}	3.78×10^{5}	7.81×10^{5}
10^{16}	1.11×10^{4}	6.90×10^{4}	3.82×10^{4}	7.91×10^{4}
10^{17}	1.24×10^{3}	7.58×10^{3}	4.20×10^{3}	8.67×10^{3}
10^{18}	184	1.08×10^{3}	604	1.22×10^{3}
10^{19}	47.4	243	146	272
10^{20}	52.2	194	121	217
10^{21}	286	884	500	1.00×10^{3}

Table S26. Average hole relaxation time ($\langle\tau_{\text{total}}\rangle$) and *a*-, *b*-, and *c*-axis hole mean free path ($\langle l_{\text{total}}\rangle$) for α-$FAPbI_3$, δ-$FAPbI_3$, and $CsPbI_3$ single crystals, respectively at room temperature.

α-$FAPbI_3$				
N (cm^{-3})	$<\tau_{total}>$ (fs)	$<l_{total}>$ (Å)		
		a axis	*b* axis	*c* axis
10^{13}	7.77	9.98	9.98	47.7
10^{14}	7.77	9.98	9.98	47.7
10^{15}	7.76	9.98	9.98	47.7
10^{16}	7.73	9.96	9.96	47.6
10^{17}	7.51	9.77	9.77	46.9

10^{18}	6.89	9.21	9.21	44.2
10^{19}	6.19	8.45	8.45	39.4
10^{20}	6.15	8.18	8.18	37.9
10^{21}	7.07	9.10	9.10	43.3

δ-$FAPbI_3$

N (cm^{-3})	$<\tau_{total}>$ (fs)	$<l_{total}>$ (Å)		
		a axis	*b* axis	*c* axis
10^{13}	2.00	2.99	3.60	14.4
10^{14}	2.00	2.99	3.60	14.4
10^{15}	2.00	2.99	3.60	14.4
10^{16}	1.99	2.98	3.59	14.4
10^{17}	1.94	2.93	3.52	14.1
10^{18}	1.80	2.78	3.34	13.4
10^{19}	1.74	2.68	3.22	12.7
10^{20}	1.84	2.76	3.33	13.2
10^{21}	1.96	2.93	3.53	14.1

$CsPbI_3$

N (cm^{-3})	$<\tau_{total}>$ (fs)	$<l_{total}>$ (Å)		
		a axis	*b* axis	*c* axis
10^{13}	5.49	20.4	13.3	23.5
10^{14}	5.49	20.4	13.3	23.5
10^{15}	5.48	20.4	13.3	23.5
10^{16}	5.47	20.4	13.3	23.5
10^{17}	5.38	20.2	13.2	23.3
10^{18}	5.08	19.5	12.7	22.4
10^{19}	4.73	18.3	11.9	20.9
10^{20}	4.94	18.4	12.0	21.1
10^{21}	5.35	19.9	12.9	22.8

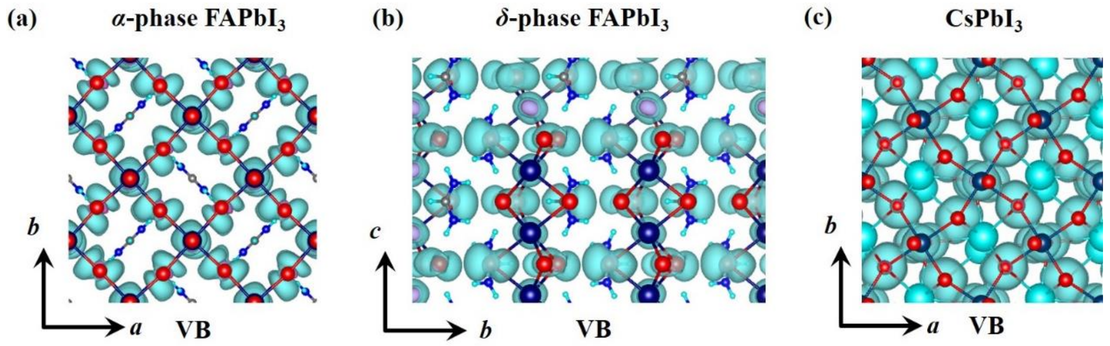

Figure S20. Charge density isosurfaces for the valence bands (VB) for crystalline (**a**) α-$FAPbI_3$, (**b**) δ-$FAPbI_3$, and (**c**) $CsPbI_3$, respectively. The lead and iodine atoms are shown in dark blue and red, respectively.

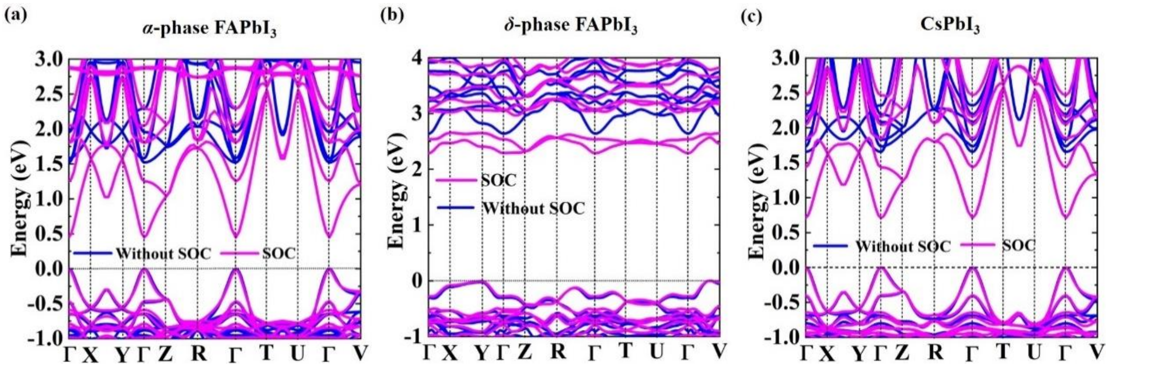

Figure S21. Calculated band structures without spin-orbital coupling (SOC, blue) and including SOC (pink) for crystalline (**a**) α-$FAPbI_3$, (**b**) δ-$FAPbI_3$, and (**c**) $CsPbI_3$, respectively. It is proven that the SOC has negligible influence on the valence band structures for our studied materials. The band energies are shifted relative to the valence band maximum. The Fermi energy is highlighted in horizontal black dashed line. The reciprocal coordinates of high symmetry *k*-points in the first Brillouin zone are Γ = (0, 0, 0), X = (0.5, 0, 0), Y = (0, 0.5, 0), Z = (0, 0, 0.5), R = (0.5, 0.5, 0.5), T = (0, 0.5, 0.5), U = (0.5, 0, 0.5), and V = (0.5, 0.5, 0), respectively.

Table S27. Band gap, and hole effective mass (m^*/m_0) for α-$FAPbI_3$, δ-$FAPbI_3$, and $CsPbI_3$ single crystals, respectively at room temperature. Here, the effective mass is defined as $\left(\frac{1}{m^*}\right)_{\alpha\beta} = \left(\frac{1}{\hbar^2}\right)\left(\frac{\partial^2 E}{\partial k_\alpha \partial k_\beta}\right)$.

α-$FAPbI_3$	E_g (eV)	m^*/m_0						
		ΓX	ΓY	ΓZ	ΓR	ΓT	ΓU	ΓV
	1.52	0.22	0.22	0.14	0.26	0.19	0.19	0.33
δ-$FAPbI_3$	E_g (eV)	m^*/m_0						
		XΓ	YX	YΓ		ΓV	VΓ	
	2.64	1.2	1.2	0.53		1.26	1.87	
$CsPbI_3$	E_g (eV)	m^*/m_0						
		ΓX	ΓY	ΓZ	ΓR	ΓT	ΓU	ΓV
	1.66	0.24	0.29	0.23	0.27	0.26	0.24	0.28

Section S6. Supporting References

[1] P. E. Blöchl, *Phys. Rev. B* **1994**, *50*, 17953.
[2] J. P. Perdew, K. Burke, M. Ernzerhof, *Phys. Rev. Lett.* **1996**, *77*, 3865.
[3] S. Grimme, J. Antony, S. Ehrlich, H. Krieg, *J. Chem. Phys.* **2010**, *132*, 154104.
[4] G. Kresse, J. Furthmüller, *Comput. Mater. Sci.* **1996**, *6*, 15.
[5] Q. Han, S.-H. Bae, P. Sun, Y.-T. Hsieh, Y. (Michael) Yang, Y. S. Rim, H. Zhao, Q. Chen, W. Shi, G. Li, Y. Yang, *Adv. Mater.* **2016**, *28*, 2253.
[6] B. Zhao, S.-F. Jin, S. Huang, N. Liu, J.-Y. Ma, D.-J. Xue, Q. Han, J. Ding, Q.-Q. Ge, Y. Feng, J.-S. Hu, *J. Am. Chem. Soc.* **2018**, *140*, 11716.
[7] J. S. Tse, N. J. English, K. Yin, T. Iitaka, *J. Phys. Chem. C* **2018**, *122*, 10682.
[8] D. Wang, L. Tang, M. Long, Z. Shuai, *J. Phys. Chem. C* **2011**, *115*, 5940.

[9] F. Bottin, J. Bieder, J. Bouchet, *Comput. Phys. Commun.* **2020**, *254*, 107301.
[10] W. Shi, E. Yildirim, G. Wu, Z. M. Wong, T. Deng, J.-S. Wang, J. Xu, S.-W. Yang, *Adv. Theory Simul.* **2020**, *3*, 2000015.
[11] C. M. Iaru, A. Brodu, N. J. J. van Hoof, S. E. T. ter Huurne, J. Buhot, F. Montanarella, S. Buhbut, P. C. M. Christianen, D. Vanmaekelbergh, C. de Mello Donega, J. G. Rivas, P. M. Koenraad, A. Yu. Silov, *Nat. Commun.* **2021**, *12*, 5844.
[12] A. D. Wright, C. Verdi, R. L. Milot, G. E. Eperon, M. A. Pérez-Osorio, H. J. Snaith, F. Giustino, M. B. Johnston, L. M. Herz, *Nat. Commun.* **2016**, *7*, 11755.
[13] R. P. Feynman, *Phys. Rev.* **1955**, *97*, 660.
[14] R. P. Feynman, R. W. Hellwarth, C. K. Iddings, P. M. Platzman, *Phys. Rev.* **1962**, *127*, 1004.
[15] M. Gajdoıfmmode \checks\else š\fi, K. Hummer, G. Kresse, J. Furthmüller, F. Bechstedt, *Phys. Rev. B* **2006**, *73*, 045112.
[16] W. Shi, Z. M. Wong, T. Deng, G. Wu, S.-W. Yang, *Adv. Funct. Mater.* **2021**, *31*, 2007438.
[17] W. Shi, G. Wu, K. Hippalgaonkar, J.-S. Wang, J. Xu, S.-W. Yang, *J. Am. Chem. Soc.* **2018**, *140*, 13200.
[18] T. Zhao, W. Shi, J. Xi, D. Wang, Z. Shuai, *Sci. Rep.* **2016**, *6*, 19968.
[19] Y. Wang, Y. Zhang, P. Zhang, W. Zhang, *Phys. Chem. Chem. Phys.* **2015**, *17*, 11516.
[20] J. Bardeen, W. Shockley, *Phys. Rev.* **1950**, *80*, 72.
[21] W. Shi, J. Chen, J. Xi, D. Wang, Z. Shuai, *Chem. Mater.* **2014**, *26*, 2669.
[22] S.-H. Wei, A. Zunger, *Phys. Rev. B* **1999**, *60*, 5404.
[23] W. Shi, T. Zhao, J. Xi, D. Wang, Z. Shuai, *J. Am. Chem. Soc.* **2015**, *137*, 12929.
[24] T. Zhao, D. Wang, Z. Shuai, *Synth. Met.* **2017**, *225*, 108.
[25] X. Mettan, R. Pisoni, P. Matus, A. Pisoni, J. Jaćimović, B. Náfrádi, M. Spina, D. Pavuna, L. Forró, E. Horváth, *J. Phys. Chem. C* **2015**, *119*, 11506.
[26] S.-N. Hsu, W. Zhao, Y. Gao, Akriti, M. Segovia, X. Xu, B. W. Boudouris, L. Dou, *Nano Lett.* **2021**, *21*, 7839.
[27] W. Lee, H. Li, A. B. Wong, D. Zhang, M. Lai, Y. Yu, Q. Kong, E. Lin, J. J. Urban, J. C. Grossman, P. Yang, *Proc. Natl. Acad. Sci.* **2017**, *114*, 8693.
[28] T. Liu, X. Zhao, J. Li, Z. Liu, F. Liscio, S. Milita, B. C. Schroeder, O. Fenwick, *Nat. Commun.* **2019**, *10*, 5750.
[29] L. Wang, Z. Zhang, Y. Liu, B. Wang, L. Fang, J. Qiu, K. Zhang, S. Wang, *Nat. Commun.* **2018**, *9*, 3817.
[30] Y. Sun, L. Qiu, L. Tang, H. Geng, H. Wang, F. Zhang, D. Huang, W. Xu, P. Yue, Y. Guan, F. Jiao, Y. Sun, D. Tang, C. Di, Y. Yi, D. Zhu, *Adv. Mater.* **2016**, *28*, 3351.
[31] A. Pisoni, J. Jaćimović, O. S. Barišić, M. Spina, R. Gaál, L. Forró, E. Horváth, *J. Phys. Chem. Lett.* **2014**, *5*, 2488.
[32] G. A. Elbaz, W.-L. Ong, E. A. Doud, P. Kim, D. W. Paley, X. Roy, J. A. Malen, *Nano Lett.* **2017**, *17*, 5734.
[33] R. Heiderhoff, T. Haeger, N. Pourdavoud, T. Hu, M. Al-Khafaji, A. Mayer, Y. Chen, H.-C. Scheer, T. Riedl, *J. Phys. Chem. C* **2017**, *121*, 28306.
[34] C. Ge, M. Hu, P. Wu, Q. Tan, Z. Chen, Y. Wang, J. Shi, J. Feng, *J. Phys. Chem. C* **2018**, *122*, 15973.
[35] H. Ma, C. Li, Y. Ma, H. Wang, Z. W. Rouse, Z. Zhang, C. Slebodnick, A. Alatas, S. P. Baker, J. J. Urban, Z. Tian, *Phys. Rev. Lett.* **2019**, *123*, 155901.
[36] T. Haeger, M. Wilmes, R. Heiderhoff, T. Riedl, *J. Phys. Chem. Lett.* **2019**, *10*, 3019.
[37] Y. Le Page, P. Saxe, *Phys. Rev. B* **2002**, *65*, 104104.
[38] A. Togo, I. Tanaka, *Scr. Mater.* **2015**, *108*, 1.
[39] C. Lee, W. Yang, R. G. Parr, *Phys. Rev. B* **1988**, *37*, 785.
[40] M. J. Frisch, G. W. Trucks, H. B. Schlegel, G. E. Scuseria, M. A. Robb, J. R.

Cheeseman, G. Scalmani, V. Barone, G. A. Petersson, H. Nakatsuji, X. Li, M. Caricato, A. V. Marenich, J. Bloino, B. G. Janesko, R. Gomperts, B. Mennucci, H. P. Hratchian, J. V. Ortiz, A. F. Izmaylov, J. L. Sonnenberg, Williams, F. Ding, F. Lipparini, F. Egidi, J. Goings, B. Peng, A. Petrone, T. Henderson, D. Ranasinghe, V. G. Zakrzewski, J. Gao, N. Rega, G. Zheng, W. Liang, M. Hada, M. Ehara, K. Toyota, R. Fukuda, J. Hasegawa, M. Ishida, T. Nakajima, Y. Honda, O. Kitao, H. Nakai, T. Vreven, K. Throssell, J. A. Montgomery Jr., J. E. Peralta, F. Ogliaro, M. J. Bearpark, J. J. Heyd, E. N. Brothers, K. N. Kudin, V. N. Staroverov, T. A. Keith, R. Kobayashi, J. Normand, K. Raghavachari, A. P. Rendell, J. C. Burant, S. S. Iyengar, J. Tomasi, M. Cossi, J. M. Millam, M. Klene, C. Adamo, R. Cammi, J. W. Ochterski, R. L. Martin, K. Morokuma, O. Farkas, J. B. Foresman, D. J. Fox, **2016**.
